# The Health Benefits of Exercise: Molecular and Cellular Mechanisms

**DOI:** 10.1002/mco2.70926

**Published:** 2026-09-03

**Authors:** Peifeng Ying, Huilin Wang, Xiu Lu, Wenjuan Zhang, Zhehao Lin, Kai Wang, Shangxuan Li, Lu Chen, Mingchao Jiang, Sarkawt Hamad, Yi Wang, Jianmin Wu, Shukuan Ling

**Affiliations:** ^1^ Oujiang Laboratory (Zhejiang Lab for Regenerative Medicine Vision and Brain Health) Wenzhou Medical University Wenzhou Zhejiang China; ^2^ State Key Laboratory of Medical Proteomics Beijing Proteome Research Center National Center for Protein Sciences (Beijing) Beijing Institute of Lifeomics Beijing China; ^3^ Center For Geriatric Medicine Key Laboratory of Alzheimer's Disease of Zhejiang Province The First Affiliated Hospital and Institute of Aging Wenzhou Medical University Wenzhou Zhejiang China; ^4^ Center For Physiology and Pathophysiology Institute For Neurophysiology University of Cologne Medical Faculty and University Hospital Cologne Germany; ^5^ Department of Physical Education Renmin University of China Beijing China; ^6^ Key Laboratory of Laboratory Medicine Ministry of Education Institute of Genomic Medicine Wenzhou Medical University Wenzhou Zhejiang China

**Keywords:** exercise, extreme environments, multisystem regulation, metabolic–immune network, personalized exercise, signaling pathways

## Abstract

Exercise is a low‐cost lifestyle intervention that can prevent and alleviate various diseases. It is a potent physiological stimulus that activates conserved molecular signaling pathways. Through the coordinated integration of multiple molecules, pathways, and systems, it leads to systemic health benefits. However, most studies focus on individual systems or molecular mechanisms, lacking systematic integration of the cross‐system regulation induced by exercise. We summarize the molecular mechanisms of exercise in the musculoskeletal, cardiovascular, nervous systems, among others. Exercise induces the release of exerkines (e.g., irisin, interleukin‐6, and brain‐derived neurotrophic factor) and extracellular vesicles, which activate key signaling pathways to enhance mitochondrial function, metabolism and physiological adaptation, while suppressing inflammation and oxidative stress, thereby alleviating diseases and delaying aging through cross‐system coordination. We further explore exercise‐induced adaptive regulation in extreme environments, including microgravity, hyperbaria, and hypoxia, offering a multifaceted perspective on organismal health regulation. Finally, we outline the prospects and challenges of multiomics, artificial intelligence‐driven precision medicine, personalized exercise prescriptions, and exercise mimetics. Overall, this review provides a more integrated perspective on the molecular basis of exercise and offers directions for future mechanistic and translational studies.

## Graphical abstract

Our synthesis integrates evidence on exercise‐induced regulatory mechanisms across multiple systems (musculoskeletal, cardiovascular, nervous, metabolic and immune), cross‐system, and aging. We also discusses adaptive regulation of exercise in extreme environments, along with the prospects and challenges of multiomics and AI‐driven precision exercise medicine, and exercise mimetics.

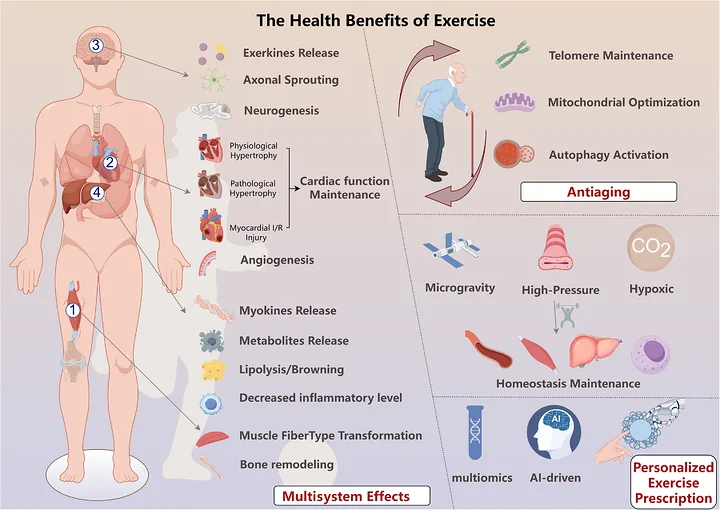

## 1 Introduction

Exercise has long been associated with improved health and reduced disease risk. With advances in epidemiology and molecular biology, its benefits are now understood to extend far beyond physical fitness, encompassing cognitive function [1], metabolic regulation [2], cardiovascular protection [3], delayed aging [4], and tissue repair and regeneration [5].

Early researches on exercise largely focused on physiological outcomes such as cardiorespiratory capacity, muscle performance, and metabolic parameters. More recently, attention has shifted toward the molecular mechanisms underlying these adaptations. Exercise is now recognized as a systemic stimulus that triggers coordinated responses across multiple tissues [6, 7]. Organs such as skeletal muscle and adipose tissue actively secrete signaling molecules, including exerkines, metabolites, and extracellular vesicle (EVs), which mediate interorgan communication and contribute to whole‐body adaptation by regulating key processes such as mitochondrial function, inflammatory responses, and metabolic homeostasis [8]. This conceptual shift has been driven by advances in high‐throughput omics approaches [9, 10, 11] (including genomics, transcriptomics, proteomics, and metabolomics), together with high‐resolution imaging [12, 13] and gene‐editing techniques [14]. These tools have enabled a more detailed dissection of the complex molecular networks and intercellular interactions elicited by exercise.

Despite these advances, several key gaps remain. Current studies have largely focused on individual systems, lacking a systematic understanding of the coordinated regulatory networks across multiple molecules and organ systems induced by exercise. How exercise achieves systemic health benefits via cross‐system regulatory mechanisms remains unclear. Furthermore, a systematic theoretical framework and translational strategy for integrating emerging technologies, including multiomics and artificial intelligence (AI), into exercise medicine are still lacking. Such integration is essential for the development of precise exercise prescriptions, personalized intervention strategies, and exercise mimetics.

To address these gaps, we integrate exercise‐induced molecular signaling pathways, cross‐system coordinated regulatory mechanisms, adaptive regulation in extreme environments, and multiomics and AI‐driven precision exercise medicine into a unified framework. Overall, this review provides a broader and deeper understanding of the systemic health benefits of exercise and offers a theoretical basis for future translational studies in precision exercise medicine.

## 2 Molecular Adaptations To Exercise Across Physiological Systems

Regular exercise serves as a pivotal strategy for maintaining and improving systemic health. By modulating the functions of multiple organ systems and fostering adaptive remodeling of tissues, exercise exerts broad beneficial effects throughout the body. These effects are mediated by a series of intracellular molecular events, involving several core signaling pathways, including adenosine monophosphate‑activated protein kinase (AMPK) [15], mechanistic target of rapamycin kinase (mTOR) [15], peroxisome proliferator‑activated receptor gamma coactivator 1‑alpha (PGC‑1α) [16], and the sirtuins family [16, 17]. These pathways sense cellular energy demands, oxidative stress, and mechanical strain, transducing such signals into intracellular responses that orchestrate physiological homeostasis. Dissection of these molecular signaling pathways enables systematic elucidation of the adaptive mechanisms underlying exercise‑induced adaptations across the musculoskeletal, cardiovascular, neural, immune, and metabolic systems [18]. Furthermore, studies on exerkines have deepened our understanding of intertissue crosstalk and revealed how exercise‐regulated molecular networks contribute to the systemic benefits of physical activity [19]. Here, we summarize the molecular adaptations induced by exercise in major physiological systems and discuss how these responses contribute to systemic homeostasis. We also highlight current limitations and areas that require further investigation, particularly regarding intersystem coordination and translational applications. Overall, exercise represents a cornerstone of nonpharmacological intervention and one of the most accessible strategies for health promotion.

### 2.1 Exercise Modulates Musculoskeletal Health

The musculoskeletal system provides a physical scaffold [20, 21], where muscles and bones mutually support each other to sustain body movement [22, 23]. Exercise regulates this system through mechanical loading, energy metabolism, and hormone secretion. Moreover, through the secretion of myokines and osteokines, exercise modulates the signaling networks involved in bone remodeling and muscle protein synthesis, thereby promoting bidirectional muscle–bone crosstalk [24]. This coordination facilitates adaptive responses in both tissues and establishes a positive feedback loop, collectively counteracting degenerative diseases such as osteoporosis and sarcopenia.

#### 2.1.1 Effects of Exercise on Skeletal Health

##### 2.1.1.1 Key Signaling Networks of Bone Metabolism Regulated by Exercise

**FIGURE 1:**
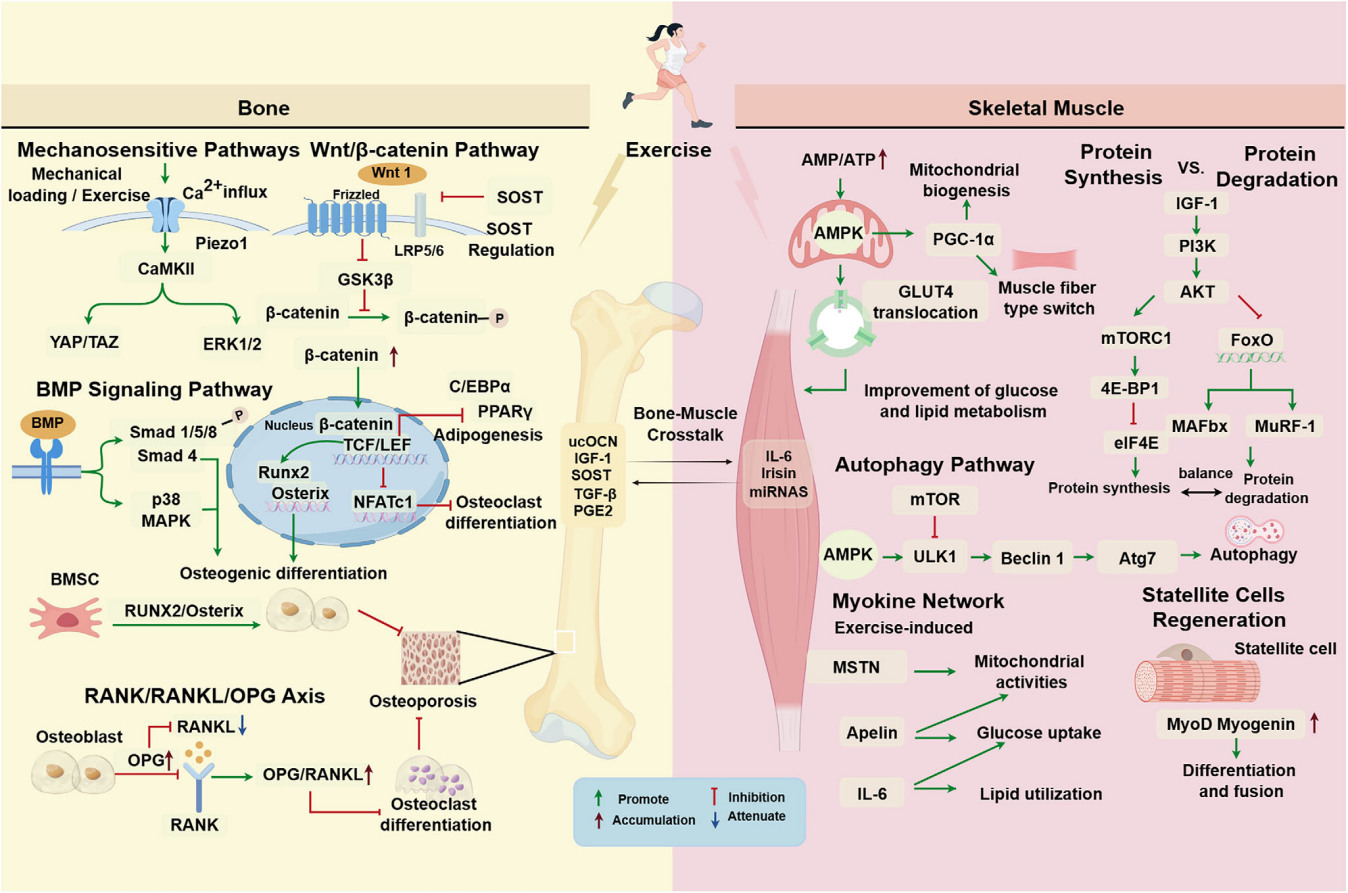
Molecular mechanisms of exercise‐induced adaptations in skeletal muscle and bone. In bone, mechanical loading activates Piezo1 and downstream Ca^2^^+^/calmodulin‐dependent protein kinase II (CaMKII), Yes‐associated protein/transcriptional coactivator with PDZ‐binding motif (YAP/TAZ), and extracellular signal‐regulated kinase 1/2 (ERK1/2) signaling. Exercise also reduces sclerostin (SOST)‐mediated inhibition of low‐density lipoprotein receptor‐related protein 5/6 (LRP5/6), activating wingless‑related integration site (Wnt)/β‐catenin signaling. Nuclear β‐catenin binds T‐cell factor/lymphoid enhancer factor (TCF/LEF) and induces Runt‐related transcription factor 2 (Runx2) and Osterix to promote osteogenesis, while suppressing nuclear factor of activated T cells cytoplasmic 1 (NFATc1)‐dependent osteoclast differentiation. Exercise further reduces adipogenic differentiation of bone marrow mesenchymal stem cells (BMSCs) by inhibiting CCAAT/enhancer‐binding protein alpha (C/EBPα) and peroxisome proliferator‐activated receptor gamma (PPARγ). Bone morphogenetic proteins (BMPs) bind to receptors, phosphorylate Smad family member 1/5/8 (Smad1/5/8), form complexes with Smad4, and activate osteogenic gene transcription. BMPs also activate noncanonical p38 mitogen‐activated protein kinase (p38 MAPK) to coregulate osteogenic gene expression, effectively stimulating bone formation. Exercise also increases osteoprotegerin (OPG) and decreases receptor activator of nuclear factor‑kappa B ligand (RANKL), thereby limiting RANKL/RANK‐mediated osteoclastogenesis and bone resorption. In skeletal muscle, exercise increases ATP consumption and the AMP/ATP ratio, activating AMP‐activated protein kinase (AMPK), which upregulates peroxisome proliferator‐activated receptor gamma coactivator 1‐alpha (PGC‐1α), regulates mitochondrial biogenesis and glucose transporter Type 4 (GLUT4) translocation, and promotes oxidative myofiber conversion. Exercise upregulates insulin‐like growth factor 1 (IGF‐1), activating the phosphoinositide 3‐kinase (PI3K)/protein kinase B (AKT) pathway. AKT activates mechanistic target of rapamycin complex 1 (mTORC1), relieving eukaryotic translation initiation factor 4E‐binding protein 1 (4E‐BP1)‐mediated inhibition of eukaryotic translation initiation factor 4E (eIF4E) to promote protein synthesis. Simultaneously, AKT inhibits forkhead box O (FoxO), downregulating ubiquitin ligases such as muscle atrophy F‐box (MAFbx) and muscle RING‐finger protein‐1 (MuRF‐1), thereby reducing protein degradation. AMPK also activates UNC‐51‐like kinase 1 (ULK1) and antagonizes mTORC1‐mediated ULK1 inhibition, inducing Beclin1‐and autophagy‐related 7 (Atg7)‐dependent autophagy. Exercise further activates satellite cells, increases myoblast determination protein 1 (MyoD) and myogenin, and promotes muscle repair. Exercise‐induced interleukin‐6 (IL‐6) promotes muscle hypertrophy, lipolysis, and glucose uptake, whereas myostatin negatively regulates muscle mass. Bone–muscle crosstalk is mediated by bone‐derived factors, including uncarboxylated osteocalcin (ucOCN), IGF‐1, SOST, transforming growth factor beta (TGF‐β), and prostaglandin E2 (PGE2), as well as myokines such as interleukin‐6 (IL‐6) and irisin and miRNAs.

Bone is a highly plastic tissue capable of adapting to external mechanical loading stimuli. Mechanical stimulation generated by exercise drives adaptive bone remodeling through a complex signal network. The mechanosensitive ion channel Piezo1 serves as a core regulator of bone homeostasis [25]. It senses mechanical loading and transduces this stimulus into calcium influx, thereby activating downstream signaling pathways including Ca^2^^+^/calmodulin‐dependent protein kinase II (CaMKII), yes‐associated protein and transcriptional coactivator with PDZ‐binding motif (YAP/TAZ), and extracellular signal‐regulated kinase 1/2 (ERK1/2) [26, 27, 28]. Exercise further enhances Piezo1‐mediated Ca^2^^+^ influx and phosphorylated protein kinase B (pAKT) via cyclic loading, thus promoting osteogenic differentiation and skeletal muscle protein synthesis [29, 30]. The wingless‑related integration site (Wnt)/β‐catenin pathway is another key mechanism underlying exercise‐induced bone formation [31]. Mechanical stimulation activates Wnt/β‐catenin signaling, promotes runt‐related transcription factor 2 (Runx2) and Osterix expression, enhances osteogenic differentiation of bone marrow mesenchymal stem cells (BMSCs), and inhibits osteoclast differentiation [31, 32]. This pathway also reduces adipogenic differentiation of BMSCs and favors osteogenic commitment by repressing CCAAT/enhancer‑binding protein alpha (C/EBPα) and peroxisome proliferator‐activated receptor gamma (PPARγ) [33, 34]. Furthermore, epigenomic regulation triggered by exercise inhibits the expression of sclerostin (SOST), relieves the suppression of low‑density lipoprotein receptor‑related protein 5/6 (LRP5/6), sustains Wnt pathway activity, and promotes bone formation [35, 36] (Figure 1).

The bone morphogenetic protein (BMP) signaling pathway and the receptor activator of nuclear factor‑kappa B (RANK)/RANKL/osteoprotegerin (OPG) signaling axis cooperatively maintain bone homeostasis by promoting bone formation and regulating bone metabolism. During exercise, BMPs orchestrate the differentiation of BMSCs through both suppressor of mothers against decapentaplegic (Smad)‑dependent and p38 mitogen‐activated protein kinase (p38 MAPK) pathways [37]. Studies have demonstrated that 5 weeks of treadmill exercise increases the expression of osteocalcin (OCN) and osteopontin (OPN), activates the BMP/Smad signaling, stimulates bone formation, and increases bone mineral density (BMD) [38, 39]. The RANK/RANKL/OPG signaling axis precisely controls the balance between bone resorption and bone formation [40]. Exercise inhibits bone resorption by upregulating OPG expression and blocking the RANK/RANKL interaction, thereby preserving bone mass [40]. As a decoy receptor, OPG competitively binds RANKL and prevents osteoclast differentiation [41]. There is an evidence that whole‑body vibration training promotes bone formation and suppresses bone resorption by maintaining osteogenic markers and reducing RANKL expression, thereby improving BMD in postmenopausal women with osteoporosis [42] (Figure 1).

##### 2.1.1.2 Regulation of Bone‐Related Diseases by Exercise

Exercise is an effective nonpharmacological intervention for preventing and treating various bone diseases, primarily by restoring bone metabolic homeostasis through the regulation of multiple signaling pathways [43, 44]. Exercise alleviates age‐related decline in skeletal mechanosensitivity caused by Piezo1 dysfunction, thereby ameliorating senile and disuse osteoporosis [45]. Although Piezo1 agonists can restore bone mass in osteoporosis models by reactivating mechanotransduction [46], excessive activation leads to Ca^2+^ overload and chondrocyte apoptosis [46, 47]. In postmenopausal osteoporosis, estrogen deficiency upregulates RANKL expression, whereas exercise increases bone density and reduces fracture risk [48]. In senile osteoporosis, endurance and resistance training mitigate age‐related bone loss by improving mitochondrial biogenesis and suppressing oxidative stress [49]. Additionally, acute exercise stimulates skeletal muscle to secrete interleukin‐6 (IL‐6), which may promote M2 macrophage polarization during bone repair and thereby create a pro‐osteogenic microenvironment [50, 51, 52]. Exercise‐induced irisin released from skeletal muscle can bind to integrin αV/β5 receptors and enhance osteocyte survival [53, 54]. Collectively, exercise serves as a fundamental nonpharmacological and individualized therapeutic strategy for multiple bone diseases.

#### 2.1.2 Regulation of Skeletal Muscle Homeostasis by Exercise

##### 2.1.2.1 Molecular Mechanisms Underlying Exercise‑Regulated Skeletal Muscle Homeostasis

Skeletal muscle is a primary site of metabolic regulation and a key mediator of exercise‐induced adaptations [55]. Exercise maintains skeletal muscle homeostasis through multiple mechanisms, among which mitochondrial regulation is the most central [56]. Exercise improves mitochondrial function and reduces reactive oxygen species (ROS) levels, thereby alleviating muscle atrophy. Exercise rapidly elevates AMP/ATP ratio to activate AMPK [57] and upregulate PGC‐1α [58], then regulating mitochondrial biogenesis and oxidative myofiber transition. Also, the AMPK/PGC‐1α axis enhances aerobic metabolic capacity and fatigue resistance in skeletal muscle [59] and further supports skeletal muscle health by improving insulin sensitivity, facilitating glucose transporter Type 4 (GLUT4) translocation, and promoting glucose and lipid metabolism [60]. Furthermore, exercise increases irisin levels in skeletal muscle, which enhances metabolism in myocytes and adipocytes, improves mitochondrial content and dynamics, and protects skeletal muscle [61]. Exercise also helps restore apelin levels in aging myocytes, which may protect skeletal muscle by promoting mitochondrial biogenesis, reducing inflammation, and activating muscle stem cells [62, 63].

In addition to metabolic regulation, exercise also influences protein turnover in skeletal muscle [64, 65]. Exercise enhances insulin‐like growth factor 1 (IGF‐1), activating the phosphatidylinositol 3‑kinase (PI3K)/AKT pathway and subsequently mTORC1. Activation of mTORC1 relieves the inhibitory effect of eukaryotic translation initiation factor 4E‑binding protein 1 (4E‐BP1) on eukaryotic translation initiation factor 4E (eIF4E), promotes ribosome biogenesis and protein synthesis, while inhibiting forkhead box O (FoxO)‐dependent expression of atrophy‐related ubiquitin ligases such as muscle atrophy F‐box protein (MAFbx) and muscle RING‐finger protein‐1 (MuRF‐1) [66, 67]. Proper autophagy is essential for maintaining muscle integrity and function. AMPK supports this process by activating unc‑51 like autophagy activating kinase 1 (ULK1) and opposing mTORC1‐mediated ULK1 inhibition [68]. In ovariectomized rats, 8 weeks of swimming training restored key autophagy regulators, including mTOR, ULK1, Beclin1, and autophagy‑related protein 7 (ATG7), and alleviated estrogen deficiency‐induced muscle atrophy [69].

Muscle regeneration also depends on satellite cells, the resident stem cells of skeletal muscle [70, 71]. Exercise activates satellite cells and upregulates myogenic factors such as myoblast determination protein 1 (MyoD) and myogenin, promoting satellite cell differentiation, myofiber fusion, and muscle repair [72, 73, 74]. It also improves the satellite cell microenvironment through epigenetic regulation, including methyltransferase‐like 3 (Mettl3)‐mediated N^6^‐methyladenosine modification of MyoD mRNA, and extracellular matrix remodeling [75, 76]. Furthermore, skeletal muscle acts as an endocrine organ and releases exercise–responsive myokines, including IL‐6 and myostatin (MSTN), to regulate muscle health [77, 78]. IL‐6 has been associated with exercise‐induced muscle hypertrophy [79, 80, 81], lipolysis, and glucose uptake in skeletal muscle [82]. Together, exercise supports skeletal muscle health by coordinating protein metabolism, satellite cell‐mediated regeneration, and myokine signaling (Figure 1).

##### 2.1.2.2 Regulation of Skeletal Muscle‑Related Diseases by Exercise

Exercise benefits a broad range of skeletal muscle‐related diseases by improving muscle metabolism, repair, inflammatory balance, and neuromuscular function [8]. Regular exercise promotes satellite cell proliferation and differentiation, supports myofiber regeneration, and reduces muscle atrophy [83, 84]. It also improves mitochondrial function and energy metabolism, relieving muscle weakness and wasting in degenerative disorders such as muscular dystrophy and sarcopenia [43, 85]. In traumatic muscle injury, appropriate exercise enhances local blood flow, accelerates inflammatory mediator clearance, supports collagen synthesis, and promotes functional recovery [86]. For muscle complications related to metabolic disorders or neuropathy, exercise improves the inflammatory microenvironment and neuromuscular innervation, thus reducing myalgia, stiffness, and motor dysfunction [87, 88, 89]. By strengthening antioxidant defenses and limiting oxidative stress‐induced muscle injury [90], exercise may help slow disease progression and improve motor function and quality of life.

#### 2.1.3 Bidirectional Crosstalk Network Between Muscle and Bone

Muscle and bone adapt to exercise through coordinated crosstalk. Exercise‐induced myokine‐containing exosomes from muscle act on osteocytes, while bone‐derived factors help regulate muscle homeostasis [91, 92, 93]. Together, they form a dynamic balance network that maintains musculoskeletal health.

##### 2.1.3.1 Exercise‑Induced Skeletal Muscle Exosomes in Bone Metabolic Regulation

Exercise alters the secretion and function of skeletal muscle‐derived exosomes. Combined aerobic and resistance training increases exosome‐related gene expression and promotes exosome release [94]. Exercise‑induced exosomes carry irisin, which binds to integrin αV/β5 [54], activates ERK1/2 [53] and p38 MAPK [95] signaling pathways, regulates the expression of osteogenic factors via activating transcription factor 4, maintains Type I collagen synthesis [96], optimizes osteocyte energy metabolism [97], and bidirectionally modulates bone formation and resorption to preserve skeletal homeostasis. Exercise also reshapes the exosomal microRNA (miRNA) profile to support anabolic responses in muscle and bone [98, 99]. For example, exosomes enriched with miR‐218 and miR‐27a‐3p enhance osteogenic differentiation and bone formation [24].

##### 2.1.3.2 Retrograde Regulation by Osteokines

The skeleton functions not only as a structural organ but also as an endocrine tissue that influences skeletal muscle through the secretion of osteokines, providing an important basis for bone–muscle communication [100]. Uncarboxylated OCN (ucOCN) promotes glucose and fatty acid utilization in muscle and enhances exercise adaptation [93]. Bone‐derived IGF‐1 contributes to muscle mass and postinjury repair [101, 102]. Exercise also downregulates SOST to preserve muscle function [103, 104]. Excessive release of transforming growth factor beta (TGF‐β) during bone resorption impairs muscle contractility, while its inhibition improves muscle strength [105]. Prostaglandin E2 (PGE2), released through connexin 43 hemichannels, promotes myogenesis [106, 107]. Collectively, these bone‐derived factors regulate muscle metabolism, growth, and regeneration, highlighting the endocrine role of bone in musculoskeletal homeostasis.

##### 2.1.3.3 Epigenetic Coregulation by Exercise

Beyond secreted factors, exercise coordinate muscle–bone interactions through epigenetic mechanisms. Exercise modulates the levels of metabolic intermediates and intracellular acetyl‐CoA [108], leading to changes in DNA methylation and histone acetylation that drive muscle and bone remodeling [109]. In skeletal muscle, exercise promotes hypomethylation of genes such as PGC‐1α and pyruvate dehydrogenase kinase 4 [110, 111] and modifies histone marks including acetylation of histone H3 at lysine 36 and trimethylation of histone H3 at lysine 27 [112, 113, 114], thereby enhancing mitochondrial biogenesis, fiber‐type remodeling, and regeneration [110, 111]. Mechanical loading generated by exercise downregulates DNA methyltransferase expression, leading to hypomethylation of osteogenic gene promoters (e.g., Runx2 and Bmp2) [115] and hypermethylation of the negative regulator SOST [110], which favor bone formation. Exercise also remodels exosomal miRNA profiles [24] and noncoding RNAs to modulate pathways including Wnt/β‐catenin and TGF‐β signaling [116]. Overall, epigenetic mechanisms provide an important link between metabolic and mechanical signals during exercise.

#### 2.1.4 Potential Adverse Effects of Exercise on the Musculoskeletal System

Moderate exercise improves bone density and muscle function. However, excessive high‐intensity exercise may have adverse effects on the musculoskeletal system. It induces high oxidative stress that damages bone [117], and mechanical loads beyond physiological ranges can lead to the accumulation of bone microcracks, predisposing to stress fractures [118, 119]. Excessive exercise also activates poly(ADP‐ribose) polymerase 1 and disrupts protein phosphorylation homeostasis, resulting in muscle dysfunction [120, 121]. Moreover, prolonged high‐intensity exercise causes sustained elevation of cortisol, which suppresses immunity, satellite cell activation, and protein synthesis, leading to overtraining syndrome [122, 123, 124]. These findings highlight that individualized exercise regimens that follow a progressive, diversified training approach are key strategies for preventing exercise‐induced injuries.

### 2.2 Exercise‑Mediated Regulation of Cardiovascular System Health

Cardiovascular disease (CVD) remains the leading cause of death worldwide, and its global burden continues to increase [125]. Regular exercise is a safe and effective nonpharmacological strategy that lowers both CVD incidence and mortality [126].

#### 2.2.1 Effects of Exercise on Cardiac Health

##### 2.2.1.1 Molecular Mechanisms Underlying Exercise‑Induced Cardioprotection

Exercise promotes physiological cardiac remodeling through several conserved signaling pathways. In canonical pathways, exercise‐induced IGF‑1 [127] activates PI3K/AKT signaling in cardiomyocytes, supporting cell growth, survival, and resistance to apoptosis [128, 129]. Concurrently, exercise upregulates the expression of neuregulin‑1 (NRG1). NRG1 binds to erythroblastic oncogene B 3 (ErbB3)/ErbB4 receptors on adjacent cardiomyocytes, inducing the formation of ErbB2 heterodimers, which further activates the PI3K/AKT pathway and coordinates cardiomyocyte differentiation and hypertrophic growth [130]. At the transcriptional level, reduced C/EBPβ and increased CBP/p300‑interacting transactivator 4 (CITED4) expression contribute to adaptive cardiac growth and protection against pathological remodeling and myocardial ischemia/reperfusion (MI/R) injury [131]. Exercise suppresses Hippo signaling, enabling YAP nuclear translocation, which inhibits proapoptotic and fibrotic signals and enhances cardiomyocyte survival and proliferation [132, 133]. Furthermore, several exercise–responsive noncoding RNAs, including miR‐222, miR‐17‐3p, cardiomyocyte proliferation‑associated long noncoding RNA (CPhar), and exercise‑associated cardiac transcript 1 (ExACT1), also participate in regulating physiological hypertrophy [134].

**FIGURE 2:**
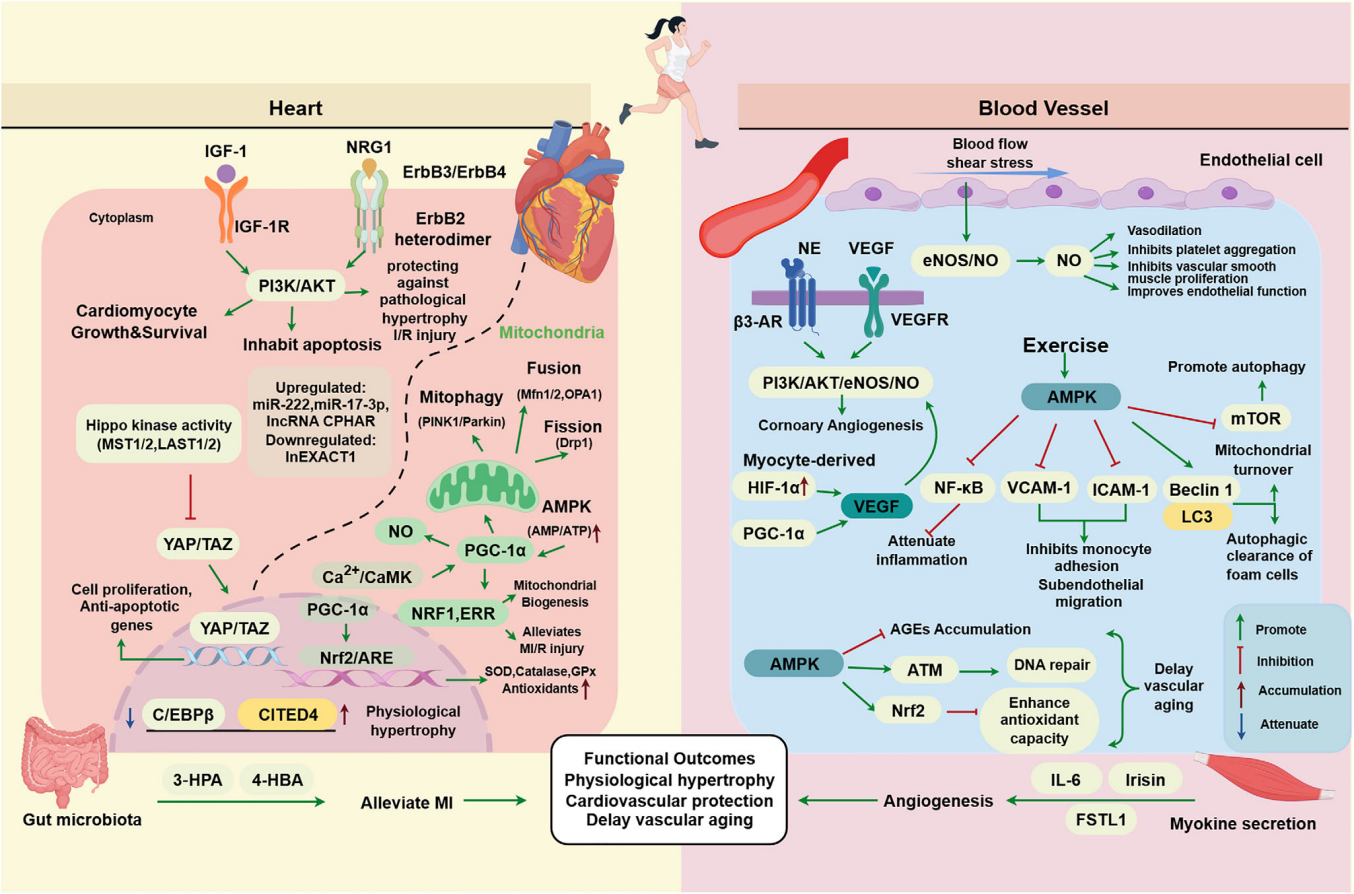
Molecular mechanisms underlying exercise‐induced cardioprotection and vascular adaptation. In the heart, exercise promotes insulin‐like growth factor 1 (IGF‐1) and neuregulin‐1 (NRG1) signaling, activates phosphoinositide 3‐kinase (PI3K)/protein kinase B (Akt), and supports cardiomyocyte growth and survival. Exercise also inhibits Hippo kinase activity, promotes nuclear translocation of Yes‐associated protein/transcriptional coactivator with PDZ‐binding motif (YAP/TAZ), and maintains physiological cardiac hypertrophy. At the regulatory RNA level, exercise increases microRNA‐222 (miR‐222), miR‐17‐3p, and the cardioprotective long noncoding RNA CPhar, while reducing exercise‐associated cardiac transcript 1 (lncRNA ExACT1). It also downregulates CCAAT/enhancer‐binding protein beta (C/EBPβ) and upregulates CBP/p300‐interacting transactivator with ED‐rich tail 4 (CITED4), thereby limiting pathological hypertrophy. AMP‐activated protein kinase (AMPK) and Ca^2^^+^/calmodulin‐dependent protein kinase (Ca^2^^+^/CaMK), leading to peroxisome proliferator‐activated receptor gamma coactivator 1‐alpha (PGC‐1α)‐dependent mitochondrial biogenesis. PGC‐1α works with nuclear respiratory factors 1 (NRF1) and estrogen‐related receptor (ERR), while mitochondrial dynamics are regulated through mitofusin 1/2 (MFN1/2), optic atrophy 1 (OPA1), and dynamin‐related protein 1 (Drp1). Exercise also activates nuclear factor erythroid 2‐related factor 2/antioxidant response element (Nrf2/ARE) signaling, increasing antioxidant enzymes such as superoxide dismutase (SOD), catalase (CAT), and glutathione peroxidase (GPx). Exercise‐induced gut microbial metabolites, including 3‐hydroxyphenylacetic acid (3‐HPA) and 4‐hydroxybenzoic acid (4‐HBA), may further reduce myocardial infarction injury through Nrf2/ARE signaling. In blood vessels, exercise‐induced shear stress activates endothelial nitric oxide synthase (eNOS)/nitric oxide (NO) signaling, promoting vasodilation and improving endothelial function. Norepinephrine (NE) and vascular endothelial growth factor (VEGF) activate β3‐adrenergic receptors and VEGF receptors (VEGFRs), respectively, further stimulating PI3K/Akt/eNOS signaling and angiogenesis. Exercise also activates AMPK, inhibits mechanistic target of rapamycin (mTOR), increases Beclin 1 and microtubule‐associated protein 1 light chain 3 (LC3), and promotes foam‐cell clearance, mitochondrial renewal, and plaque regression. Skeletal muscle‐derived myokines, including interleukin‐6 (IL‐6), follistatin‐like protein 1 (FSTL1), and irisin, also support endothelial proliferation and tube formation.

Mitochondrial adaptation is another key component of exercise‐induced cardioprotection [135, 136]. Exercise training upregulates and activates PGC‑1α through multiple mechanisms, including promoting nitric oxide (NO) production [137], enhancing Ca^2^^+^/calmodulin signaling to activate CaMKs, and stimulating AMPK. As a master regulator of mitochondrial biogenesis and energy metabolism [138], PGC‑1α cooperates with transcription factors such as nuclear respiratory factor 1 (NRF1) and estrogen‑related receptor to enhance mitochondrial biogenesis and alleviating MI/R injury [139, 140]. Exercise also preserves mitochondrial network integrity by promoting PTEN‑induced putative kinase 1 (PINK1)/Parkin‑mediated mitophagy and balancing mitochondrial fusion mediated by mitofusin 1/2 (Mfn1/2) and optic atrophy 1 (OPA1) with fission mediated by dynamin‑related protein 1 (Drp1) [141]. In parallel, moderate exercise induces mild oxidative stress response that activates nuclear factor erythroid 2‐related factor 2 (Nrf2) antioxidant response element (ARE) signaling and increases endogenous antioxidant defenses, including superoxide dismutase (SOD), catalase (CAT), and glutathione peroxidase (GPx) [142, 143]. Notably, exercise‐induced gut microbial metabolites such as 3‑hydroxyphenylacetic acid (3‑HPA) and 4‑hydroxybenzoic acid (4‑HBA) contribute to cardioprotection through Nrf2‐dependent mechanisms [144, 145, 146] (Figure 2).

##### 2.2.1.2 Regulation of Cardiac Diseases by Exercise

Exercise exerts preventive and therapeutic benefits against a spectrum of cardiovascular diseases (CVDs) [147, 148, 149]. including myocardial infarction, MI/R, diabetic cardiomyopathy, atrial fibrillation, and heart failure, by integrating multilevel mechanisms such as signal transduction [150], antioxidant defense [151, 152], energy metabolism remodeling [153], and gut microbiota regulation [146]. Studies have demonstrated that exercise activates the PI3K/AKT signaling pathway [147, 148, 149] and enhances Nrf2‐mediated antioxidant responses to restore antioxidant capacity and attenuate pressure overload‑induced cardiac remodeling [154, 155, 156]. Exercise also improves cardiac energy metabolism through PGC‐1α, PPARα, and PPARγ, which effectively alleviates diabetic cardiomyopathy and mitigates acute myocardial infarction [153, 157, 158]. In addition, exercise can regulate the microbiota and related metabolites, which may help prevent myocardial infarction [146]. Clinically, regular exercise improves myocardial perfusion, reduces ischemic burden, elevates angina threshold, and enhances cardiac reserve function in patients with coronary artery disease [159, 160]. Therefore, selecting appropriate exercise modality, intensity, frequency, and duration exhibits significant efficacy in both preventing and ameliorating multiple cardiac diseases.

#### 2.2.2 Effects of Exercise on Vascular Health

##### 2.2.2.1 Molecular Mechanisms of Exercise‐Mediated Vascular Protection

Endothelial dysfunction and arterial stiffening are major contributors to CVDs [161, 162]. Exercise activates the eNOS/NO pathway via blood flow shear stress to improve endothelial function [163, 164, 165]. Exercise‑induced norepinephrine (NE) and vascular endothelial growth factor (VEGF) bind to β3‑adrenergic receptors and VEGFRs on endothelial cells [166], respectively, activating the PI3K/AKT/eNOS/NO signaling and regulating coronary angiogenesis [167]. Exercise also upregulates cardiac hypoxia‑inducible factor 1‑alpha (HIF‐1α) and PGC‑1α to produce VEGF, which promotes endothelial cell survival, proliferation, and migration via the PI3K/AKT pathway, thereby enhancing angiogenesis [168, 169]. Additionally, exercise‐induced myokines, including IL‐6, irisin, and follistatin‑like 1 (FSTL1), support endothelial function and vascular angiogenesis [170, 171]. In terms of AMPK signaling, exercise‑activated AMPK suppresses nuclear factor kappa‐light‐chain‐enhancer of activated B cells (NF‑κB) [172, 173], enhances eNOS phosphorylation, reduces the expression of adhesion molecules (vascular cell adhesion molecule 1 [VCAM‑1], intercellular adhesion molecule 1), and inhibits monocyte adhesion and subendothelial migration [174, 175]. AMPK also activates Beclin‑1 and microtubule‑associated protein 1 light chain 3 (LC3) to promote autophagic clearance of foam cells and mitochondrial renewal [176, 177, 178], while inhibiting mTOR signaling to enhance autophagy, facilitating plaque regression [174, 179]. Moreover, AMPK reduces the accumulation of advanced glycation end products, maintains telomere homeostasis, activates the ataxia telangiectasia mutated (ATM)‑mediated DNA repair pathway, and upregulates Nrf2 antioxidant signaling, delaying vascular aging [180] (Figure 2).

##### 2.2.2.2 Modulation of Exercise on Vascular‐Related Diseases

Exercise induces selective autophagy in endothelial cells and mitochondria, which helps maintain vascular homeostasis and may contribute to atherosclerotic plaque regression [179, 181]. Exercise also activates AMPK and eNOS signaling, producing cardioprotective effects similar to ischemic preconditioning and increasing resistance to MI/R [164, 182]. In parallel, Nrf2 activation reduces oxidative stress, inflammasome activity, and proinflammatory cytokine production [183, 184, 185]. Together, these adaptations improve vascular function and slow the progression of CVD.

#### 2.2.3 Potential Adverse Effects of Exercise on the Cardiovascular System

Moderate exercise benefits cardiovascular health, but prolonged endurance exercise carries potential risks [186]. Long‑term high‑intensity endurance training increases the risk of early‑onset atrial fibrillation, particularly in male athletes [187]. Some studies also report higher coronary artery calcification and plaque burden in lifelong endurance athletes than in sedentary individuals [188]. Chronic extreme exercise can lead to “athlete's heart,” which, although physiological in its early stages, may provide a pathological basis for future adverse events [187]. Therefore, exercise prescription should be individualized to balance benefits and risks.

### 2.3 Nervous System: Plasticity and Defense Mechanisms

Exercise benefits the brain through multilevel regulation, from molecular pathways to neural networks, inducing adaptive changes in brain structure and function.

#### 2.3.1 Neurotrophic Factor Signaling and Neurogenesis

Exercise exerts both acute and long‐term effects on the nervous system. Acutely, it increases endorphins, dopamine, NE, and serotonin [189, 190, 191] while reducing cortisol during postexercise recovery, thereby helping relieve stress and improve mood [192]. With regular practice, exercise may promote structural and functional brain remodeling. It has been linked to increased brain‐derived neurotrophic factor (BDNF) expression, which supports neuronal survival, synaptic plasticity, hippocampal neurogenesis, and facilitates learning and memory processes [193, 194]. Neuroimaging studies further suggest that long‐term exercise is associated with greater hippocampal gray matter volume, increased prefrontal cortical thickness and connectivity, and improved white matter integrity [195, 196, 197]. These changes may strengthen large‐scale brain networks, including the default mode and executive control networks, thereby supporting cognitive functions such as executive control and working memory.

**FIGURE 3:**
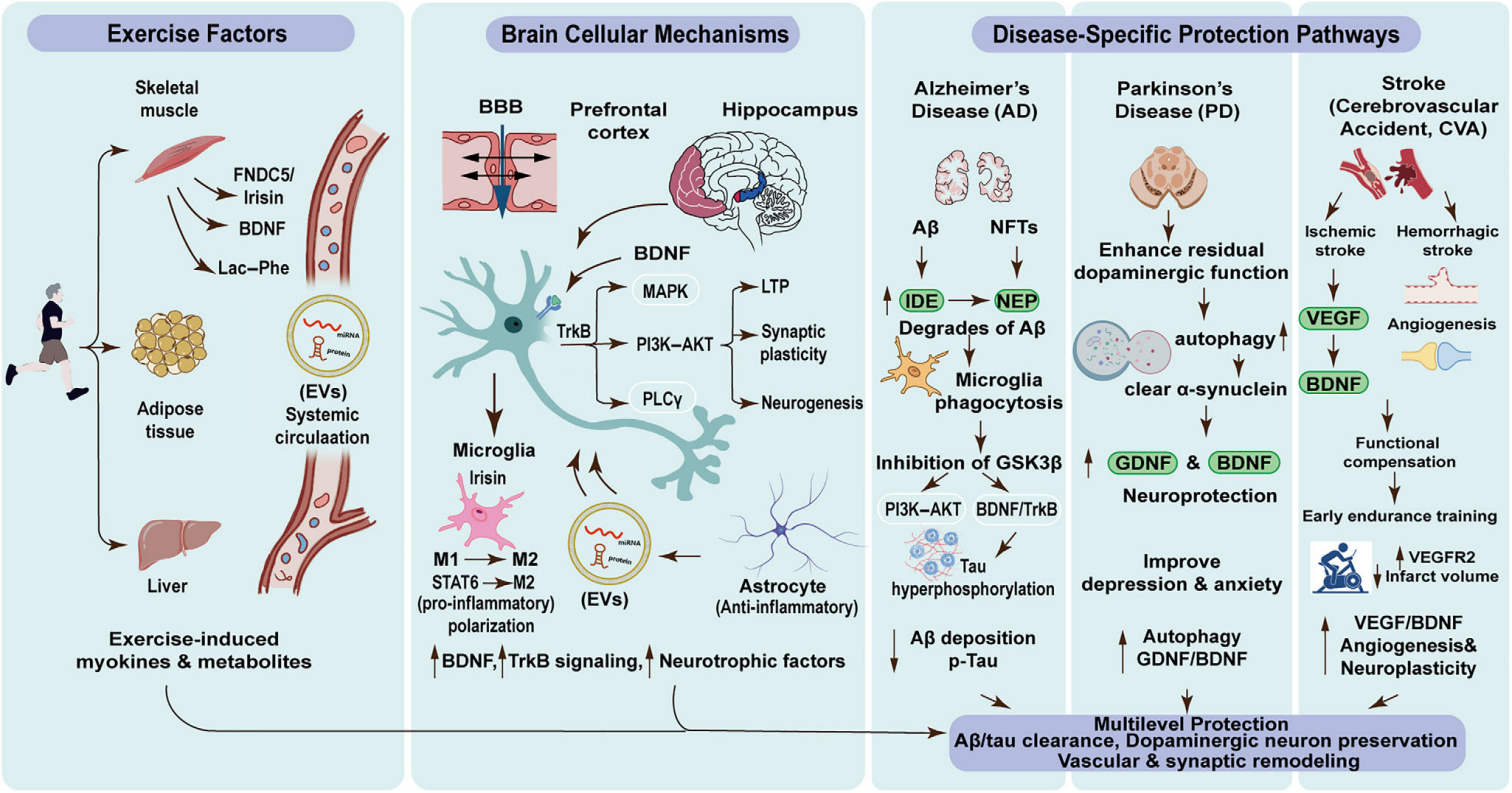
Exercise‐induced neuroplasticity and multidisease protective mechanisms. This figure summarizes how exercise supports brain health through peripheral factors, central neural mechanisms, and disease‐specific protective pathways in Alzheimer's disease (AD), Parkinson's disease (PD), and stroke/cerebrovascular accident (CVA). Exercise stimulates skeletal muscle, adipose tissue, and liver to release fibronectin Type III domain‐containing protein 5 (FNDC5)/irisin, brain‐derived neurotrophic factor (BDNF), N‐lactoyl‐phenylalanine (Lac–Phe), and extracellular vesicles (EVs), which enter the circulation and may cross the blood–brain barrier (BBB). In the brain, BDNF activates tropomyosin receptor kinase B (TrkB) and downstream mitogen‐activated protein kinase (MAPK), phosphoinositide 3‐kinase/protein kinase B (PI3K/Akt), and phospholipase C gamma (PLCγ) signaling to promote neurogenesis, synaptic plasticity, and neuronal survival. Irisin may act on microglia through signal transducer and activator of transcription 6 (STAT6) signaling, promoting an anti‐inflammatory phenotype. These effects are linked to improved structure and connectivity in the hippocampus and prefrontal cortex. In AD, exercise may enhance amyloid‐β (Aβ) clearance and reduce tau hyperphosphorylation through PI3K/Akt‐ and BDNF/TrkB‐mediated inhibition of glycogen synthase kinase‐3 beta (GSK3β), thereby limiting phosphorylated tau (p‐Tau) accumulation and neurofibrillary tangles (NFTs). In PD, exercise may support dopaminergic signaling, promote autophagic clearance of alpha‐synuclein (α‐synuclein), and increase glial cell line‐derived neurotrophic factor (GDNF) and BDNF, helping improve motor and nonmotor symptoms. In stroke/CVA, exercise increases vascular endothelial growth factor (VEGF) and BDNF, promotes angiogenesis through vascular endothelial growth factor receptor (VEGFR) signaling, reduces infarct volume, and supports functional recovery through neuroplasticity. Together, these mechanisms converge on Aβ/tau clearance, dopaminergic neuron preservation, and vascular and synaptic remodeling.

Exercise‐induced factors include proteins or peptides such as BDNF and irisin, metabolites such as lactate–phenylalanine conjugate (Lac–Phe), and EVs. Although many are secreted by contracting skeletal muscle and are often termed myokines [198], they may also arise from adipose tissue, liver, and brain [199]. These factors support neuroplasticity and neuroprotection through several mechanisms. BDNF is one of the best‐characterized mediators and is increased in the hippocampus and cortex after exercise. By binding to tropomyosin receptor kinase B (TrkB), BDNF activates MAPK, PI3K/AKT, and phospholipase C gamma (PLCγ) signaling, thereby promoting synaptic plasticity, especially long‐term potentiation (LTP), as well as neurogenesis and neuronal survival [200]. Exercise also increases skeletal muscle expression of fibronectin Type III domain‐containing 5 (FNDC5), whose cleaved extracellular domain is released as irisin. Circulating irisin promotes white adipose tissue (WAT) browning and improves systemic energy metabolism, and preclinical studies suggest that it may cross the blood–brain barrier (BBB), increase BDNF expression [201, 202], and promote an anti‐inflammatory microglial state through signal transducer and activator of transcription 6 (STAT6)‐related signaling, which may contribute to protection against postoperative cognitive dysfunction [203]. Exercise also changes the abundance and cargo of circulating EVs, including neuroactive miRNAs and proteins. These EVs can be taken up by neurons, astrocytes, and other neural cells, where they regulate gene expression, and anti‐inflammatory or neurotrophic miRNAs such as miR‐17/20a‐5p may be involved in their neuroprotective effects [204]. Furthermore, exercise may enhance astrocyte‐neuron metabolic coupling (e.g., lactate shuttling), contributing to neuronal energy support and synaptic plasticity [205] (Figure 3).

#### 2.3.2 Neuroprotective Effects Against Major Neurological Disorders

Building on these neuroplasticity mechanisms, exercise also confers protection against major neurological disorders, including Alzheimer's disease (AD), Parkinson's disease (PD), and cerebrovascular accident (CVA/stroke) are three major neurological disorders characterized by irreversible neuronal injury [206]. As a nonpharmacological strategy, exercise may exert neuroprotective effects via multitarget actions against the core pathologies of these disorders.

The core pathologies of AD are extracellular beta‐amyloid (Aβ) plaques and intracellular neurofibrillary tangles (NFTs) formed by hyperphosphorylated Tau protein [207]. Preclinical studies suggest that exercise increases insulin‐degrading enzyme and neprilysin expression and enhances microglial‐mediated Aβ clearance [208, 209]. Moreover, exercise has been associated with activation of PI3K/AKT and BDNF/TrkB signaling to inhibit glycogen synthase kinase 3 beta (GSK3β) and reduce Tau hyperphosphorylation [210].

PD is characterized by dopaminergic neuron degeneration in the substantia nigra and abnormal α‐synuclein aggregation [211, 212]. Exercise may exert neuroprotective effects by supporting residual dopaminergic signaling [213], promoting autophagy to reduce α‐synuclein aggregation, and increasing the expression of glial cell line‐derived neurotrophic factor (GDNF) and BDNF [214, 215]. It also alleviates nonmotor symptoms such as depression and anxiety through anti‐inflammatory and neurotrophic mechanisms.

Stroke is defined as brain injury caused by cerebral vessel occlusion (ischemic) or rupture (hemorrhagic) [216]. Preclinical studies suggest that exercise promotes VEGF‐related angiogenesis, improves blood supply to the ischemic penumbra [217], and increases BDNF levels to support synaptogenesis, axonal sprouting, and functional brain reorganization [209]. Animal studies further show that moderate‐intensity endurance training during the acute poststroke phase is associated with increased VEGFR2 expression and reduced infarct volume [217] (Figure 3).

#### 2.3.3 Mechanistic and Translational Challenges in Exercise Neurobiology

Despite growing interest in exercise‐induced neuroadaptation, several key challenges remain. The duration of acute exercise effects, their interindividual variability, and the dose–response relationship of BDNF are still unclear [218, 219], and exercise exerts effects on neuroinflammation dependent on intensity, timing, and neuroimmune status. Optimal exercise parameters for modulating Aβ/tau pathology [220], the long‐term impact on disease progression and nonmotor symptoms in PD, and the timing of poststroke intervention all require clarification [221]. Given the marked variability among individuals, future studies should integrate neuroimaging, biomarkers, longitudinal designs, and systems biology approaches to clarify how different exercise modalities regulate the BDNF network and neural plasticity. However, much of the current mechanistic evidence still comes from preclinical models, and stronger causal validation in humans is needed.

### 2.4 Metabolic and Immune Systems: Systemic Molecular Crosstalk

The metabolic and immune systems communicate bidirectionally to maintain organismal homeostasis [58]. Exercise reshapes this network by coordinating energy metabolism, immune signaling, and myokine release [222].

#### 2.4.1 Exercise‐Induced Myokines and Metabolic Mediators

The physiological response to exercise stems from the sensing of energy demand and mechanical stress [223]. During muscle contraction, an increased AMP/ATP ratio activates AMPK, whereas mechanical stimuli engage pathways such as mTOR that regulate metabolic and anabolic adaptation [224]. Regular exercise also reshapes the gut microbiota and its metabolites, extending exercise signaling beyond the musculoskeletal system [225, 226]. Contracting skeletal muscle releases a diverse range of myokines that contribute to the systemic effects of exercise [18]. Among them, irisin has been associated with thermogenic remodeling of adipose tissue, although its physiological relevance in humans remains uncertain [227]. Other myokines, including BDNF, IL‐15, and fibroblast growth factor 21 (FGF21), participate in the regulation of neuroplasticity, immune function, and metabolic homeostasis [228]. IL‐6 displays particularly context‐dependent effects. Acute exercise‐induced muscle‐derived IL‐6 contributes to metabolic and immunoregulatory adaptations through classical signaling pathways [229, 230, 231], whereas chronically elevated immune‐derived IL‐6 promotes inflammation through trans‐signaling [229, 232]. This dual role places IL‐6 at the interface between exercise, immunity, and metabolism [233].

**FIGURE 4:**
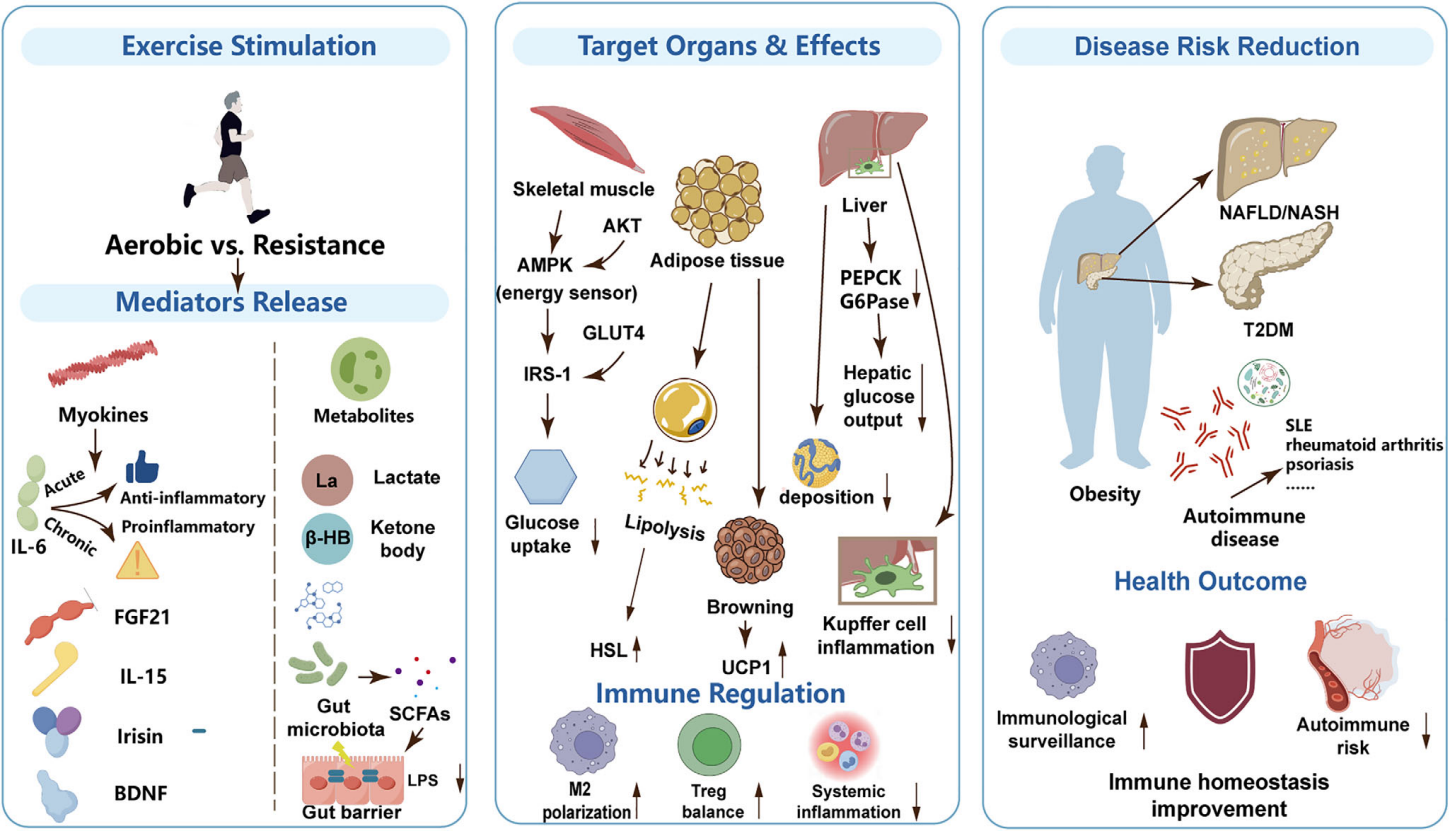
Exercise‐induced immunometabolic regulation network. This figure summarizes how exercise coordinates multiorgan crosstalk to improve metabolic and immune homeostasis. Aerobic and resistance exercise induce myokines, including interleukin‐6 (IL‐6), fibroblast growth factor 21 (FGF21), interleukin‐15 (IL‐15), irisin, and brain‐derived neurotrophic factor (BDNF), as well as metabolites such as lactate, ketone bodies, succinate, and gut microbiota‐derived short‐chain fatty acids (SCFAs). SCFAs, especially butyrate, strengthen the gut barrier, reduce lipopolysaccharide (LPS) translocation, and lower systemic inflammation. Exercise improves skeletal muscle glucose uptake by activating AMP‐activated protein kinase (AMPK) and promoting glucose transporter Type 4 (GLUT4) translocation through both insulin‐dependent insulin receptor substrate‐1/phosphoinositide 3‐kinase/protein kinase B (IRS‐1/PI3K/Akt) signaling and insulin‐independent pathways. In adipose tissue, irisin and FGF21 promote uncoupling protein 1 (UCP1)‐dependent white adipose browning, increase energy expenditure, and activate hormone‐sensitive lipase (HSL)‐mediated lipolysis. In the liver, exercise suppresses phosphoenolpyruvate carboxykinase (PEPCK) and glucose‐6‐phosphatase (G6Pase), reduces hepatic glucose output and lipid deposition, limits Kupffer cell inflammation, and promotes ketone body production, including β‐hydroxybutyrate (β‐OHB). These changes reduce systemic inflammation, support regulatory T cells (Tregs), promote macrophage M2 polarization through lactate/G protein‐coupled receptor 81 (GPR81) signaling, inhibit the NOD‐, LRR‐, and pyrin domain‐containing protein 3 (NLRP3) inflammasome through β‐OHB, and maintain gut barrier integrity. Together, these effects may reduce the risk of nonalcoholic fatty liver disease (NAFLD)/nonalcoholic steatohepatitis (NASH), Type 2 diabetes mellitus (T2DM), obesity, and immune‐mediated diseases, while improving immune homeostasis and immunological surveillance.

Exercise also alters the systemic metabolic milieu, modulating multiple circulating metabolites. Lactate acts as a dose‐dependent signaling molecule [234], physiological increases in lactate may support immune regulation, including Treg function and M2‐like macrophage polarization, partly through G protein‐coupled receptor 81 (GPR81) signaling and histone lactylation [235], whereas excessive lactate can suppress immunity through extracellular acidification and metabolic stress, especially in the tumor microenvironment [236]. Additionally, ketone bodies (e.g., β‐hydroxybutyrate) produced by the liver during exercise inhibit histone deacetylases (HDACs) and the nod‐like receptor family pyrin domain containing 3 (NLRP3) inflammasome, exerting anti‐inflammatory effects [237, 238] (Figure 4).

#### 2.4.2 Exercise–Microbiota Interactions and the Gut–Brain Axis

Beyond host‐derived metabolites, exercise also affects microbiota‐derived signals involved in systemic immunometabolic regulation. The gut microbiota is increasingly recognized as an important mediator of immunometabolic regulation [239]. Regular exercise modulates microbial composition, increasing short‐chain fatty acid (SCFA)‐producing bacteria (e.g., Faecalibacterium, Roseburia) and beneficial taxa such as Akkermansia, while modulating metabolites, including branched‐chain amino acids, tryptophan metabolites, and secondary bile acids [226, 239]. SCFAs (acetate, propionate, butyrate) exert diverse functions: butyrate enhances intestinal barrier integrity, reduces lipopolysaccharide (LPS) translocation, and lowers systemic inflammation; propionate serves as a substrate for hepatic gluconeogenesis, whereas acetate participates in peripheral lipid and energy metabolism [240, 241].

In addition to their role in gut homeostasis, SCFAs, particularly butyrate, are increasingly implicated in the exercise–microbiota–brain axis. Butyrate may cross the BBB through monocarboxylate transporter 1 (MCT1) and regulate gene expression by inhibiting HDACs [242, 243]. Preclinical studies further suggest that butyrate may influence hypothalamic appetite‐regulating pathways, including proopiomelanocortin and neuropeptide Y signaling. In addition, SCFAs may modulate neuroinflammation and help maintain hypothalamic metabolic sensitivity through free fatty acid receptor 2/3 (FFAR2/3) signaling and immune‐related mechanisms [244]. AMPK‐related signaling may also be involved, but its contribution remains unclear [245].

Clinical studies provide preliminary support for the exercise–microbiota–brain axis. Exercise interventions have been shown to alter fecal SCFA levels, accompanied by changes in energy intake and appetite‐related hormones, suggesting a role for microbial metabolites in exercise‐related feeding regulation [246]. Studies in individuals with Type 2 diabetes also indicate that exercise can modulate circulating SCFA levels. Neuroimaging studies further indicate that hypothalamic and appetite‐related brain regions are responsive to metabolic status and exercise‐related physiological changes [247, 248]. Overall, these findings support the existence of an exercise–microbiota–brain axis, but its causal relationships and molecular mechanisms require further validation in large longitudinal and mechanistic clinical studies.

#### 2.4.3 Exercise in Immunometabolic Regulation and Disease Protection

Collectively, these molecular and microbiota‐derived signals contribute to systemic immunometabolic adaptation and help prevent or modulate metabolic and inflammatory diseases. Insulin resistance, β‐cell dysfunction, and chronic low‐grade inflammation are core features of Type 2 diabetes mellitus (T2DM) and obesity [249]. Exercise exerts beneficial effects through multiorgan crosstalk, targeting adipose tissue, skeletal muscle, liver, pancreas, and the gut microbiota [18, 250].

Skeletal muscle accounts for approximately 80% of postprandial glucose disposal [251, 252]. Exercise enhances glucose uptake by promoting GLUT4 translocation through insulin receptor substrate 1 (IRS‐1)/PI3K/AKT signaling and insulin‐independent AMPK activation, and chronic training may further increase GLUT4 expression [253, 254]. Exercise also improves hepatic insulin sensitivity, suppresses gluconeogenic genes such as phosphoenolpyruvate carboxykinase (PEPCK) and glucose‐6‐phosphatase (G6Pase), lowers fasting glucose [255, 256], and reduces hepatic lipid accumulation, Kupffer cell activation, and inflammation, thereby attenuating features of nonalcoholic steatohepatitis (NASH) [257, 258]. In adipose tissue, exercise helps reduce adipocyte hypertrophy and promotes lipid mobilization through hormone‐sensitive lipase (HSL), increasing fatty acid use by skeletal muscle and reducing lipotoxic stress. Myokines such as irisin and FGF21 may further support thermogenic adipose remodeling and adaptive energy metabolism [259, 260, 261].

Various immune‐mediated diseases are linked to metabolic disturbances. Atherosclerosis, driven by lipid‐induced immune inflammation, may be improved by exercise through regulation of NLRP3 inflammasome activity, endothelial function, and oxidative stress [262, 263]. Autoimmune diseases such as rheumatoid arthritis and systemic lupus erythematosus involve immune cell metabolic reprogramming and insulin resistance [264, 265], while psoriasis and inflammatory bowel disease (IBD) are closely associated with adipose tissue inflammation and gut dysbiosis [266]. By coordinating myokines, lactate, and SCFAs, exercise may help modulate immune cell metabolism and restore gut microbial balance to alleviate these disorders [226, 266] (Figure 4).

#### 2.4.4 Challenges and Unresolved Mechanisms in Exercise Immunometabolism

Although myokines, metabolites, and the gut microbiota are increasingly recognized as key mediators of exercise‐induced metabolic–immune homeostasis, several questions remain. For example, the context‐dependent role of IL‐6 is recognized, yet its functional switching threshold and regulation by different exercise parameters are still unclear [229]. Lactate has dose‐dependent immunomodulatory effects, but physiological versus excessive levels and tissue‐specific effects require further study [267]. Exercise effects on gut microbiota are also dose dependent. Moderate training improves microbial homeostasis, while high‐intensity prolonged exercise may be detrimental. Causal links with SCFA‐producing bacteria require longitudinal validation [226, 230, 268]. Although exercise improves T2DM and obesity, the relative effects of different exercise modalities on insulin sensitivity remain to be clarified [269]. Given substantial interindividual variability, evidence for autoimmune diseases and IBD is still limited, and whether the benefits depend on specific myokine or metabolite profiles remains uncertain [270]. Future studies should integrate systems biology and integrative physiology to clarify how exercise variables influence the composition of myokine and metabolite profiles.

Thus, exercise should be viewed not merely as a means of energy expenditure, but as a systems‐level immunometabolic modulator that coordinates metabolic, immune, and microbial signaling across multiple organs, highlighting its translational potential in the prevention and management of metabolic and immune‐mediated diseases [222, 228].

### 2.5 Exercise‐Mediated Cross‐System Molecular Network

As described above, exercise activates interconnected molecular pathways that collectively form a coordinated network linking energy metabolism, inflammatory responses, and interorgan communication [58, 271]. Exerkines play a central role in this network by mediating communication between tissues through endocrine, paracrine, and autocrine pathways [272, 273].

#### 2.5.1 Irisin: A Mediator of Multisystem Adaptation

Irisin is primarily a myokine produced by skeletal muscle during exercise [274]. It promotes osteoblast differentiation by activating the p38 MAPK and c‐Jun N‐terminal kinase (JNK) pathways [95, 275] and inhibits osteoclast differentiation by suppressing the RANKL/RANK/OPG pathway [276], thereby improving osteoporosis and establishing a direct link between muscle and bone. In the cardiovascular system, irisin reduces cardiac fibrosis by activating sirtuin 1 (SIRT1) and suppressing the TGF‐β/Smad3 pathway. It also restores mitochondrial homeostasis, alleviates oxidative stress, and reduces inflammatory infiltration by enhancing the transcriptional activity of PGC‐1α and NRF1 [277]. Irisin can also cross the BBB [278], increasing cortical BDNF and pAKT levels, thereby enhancing the integrity of the neurovascular unit and the BBB [279]. In addition, irisin upregulates uncoupling protein 1 (UCP1) expression by activating the p38 MAPK and ERK pathways to promote the browning of WAT and improve energy metabolism, linking skeletal muscle activity to adipose tissue metabolic regulation [231]. These effects position irisin as a key exerkine connecting the musculoskeletal, cardiovascular, nervous, and metabolic systems.

#### 2.5.2 IL‐6: A Context‐Dependent Myokine

IL‐6, as a myokine, primarily mediates metabolic and anti‐inflammatory effects, whereas immune‐derived IL‐6 exerts proinflammatory effects, reflecting its context dependence [280]. In the musculoskeletal system, IL‐6 promotes osteoblast differentiation by activating pathways such as Janus kinase (JAK) and STAT. It also activates STAT3 signaling, stimulating osteoblasts and osteocytes to secrete RANKL, which in turn indirectly induces osteoclast differentiation. This process improves bone metabolism during exercise [51]. IL‐6 can also act on the central nervous system, promoting neuronal survival and synaptic plasticity [58]. IL‐6 facilitates glucose uptake in muscle via activation of AMPK‐ and HIF‐1α‐dependent signaling [232]. These diverse actions establish IL‐6 as an important mediator linking exercise‐induced interorgan communication.

#### 2.5.3 BDNF: A Nervous System‐Other Systems Bridge

BDNF is a key neurokine mediating brain benefits of exercise. It acts on the TrkB receptor to activate pathways such as PI3K/AKT, MAPK/ERK and PLCγ/protein kinase C (PKC), promoting dendritic growth and branching and enhancing synaptic plasticity [200, 281]. In addition, BDNF enhances hypothalamic plasticity, suppresses neuroinflammation, and improves energy metabolism and endocrine homeostasis [282]. Increased BDNF in the hippocampus promotes the MAPK/Nrf2/heme oxygenase‐1 (HO‐1) pathway, improving the antioxidant and anti‐inflammatory capacity of the liver [283]. In the heart, cardiomyocytes may sequester circulating BDNF released from the brain to activate CaMKII and AKT, enhancing cardiomyocyte contractility and increasing survival [284]. BDNF is also secreted as a myokine after exercise to promote mitochondrial biogenesis via the AMPK/PGC‐1α pathway, thus restoring exercise tolerance in mice with myocardial infarction [285, 286]. These findings on BDNF exemplify the close connection between the nervous system and other major systems in response to exercise.

#### 2.5.4 Adiponectin: A Unique Adipokine

Adiponectin is an exercise–responsive adipokine [287, 288]. Unlike most adipokines, adiponectin levels are reduced in obesity and T2DM. It plays a critical role in regulating insulin sensitivity, glucose and lipid metabolism, inflammation, and vascular protection by modulating AMPK, PPARα, and MAPK signaling [289, 290, 291]. In the cardiovascular system, exercise can reverse heart failure‐induced dysfunction of perivascular adipose tissue surrounding the thoracic aorta and improve vascular health by promoting the NE/β3‐AR/adiponectin/AMPK/eNOS signaling pathway [292]. Adiponectin signaling is not limited to the cardiovascular system but also affects skeletal muscle metabolism. Adiponectin increases glucose uptake and mitochondrial biogenesis in skeletal muscle by activating AMPK and p38 MAPK‐related pathways [231]. Furthermore, exercise‐induced adiponectin can cross the BBB and activate the AMPK–NF‐κB/STAT3 signaling pathway, thereby maintaining the M1/M2 balance of microglia and suppressing hippocampal neuroinflammation, exerting neuroprotective effects [293]. Overall, Adiponectin plays an important role in the molecular regulatory network, integrating exercise‐induced metabolic, inflammatory, and tissue remodeling responses.

#### 2.5.5 MiRNAs and EVs

MiRNAs and EVs, particularly exosomes, have recently emerged as important mediators of exercise‐induced interorgan communication [294, 295]. Adipose‐derived exosomal miR‐146a‐5p targets IGF‐1R, activates PI3K/AKT/mTOR signaling, or inhibits FoxO3 signaling, thus promoting skeletal muscle protein synthesis and ameliorating skeletal muscle atrophy [296]. Brown adipose tissue (BAT)‐derived small EVs (sEVs) deliver miR‐125b‐5p, miR‐128‐3p, and miR‐30d‐5p to the heart, suppressing proapoptotic MAPK signaling and protecting against MI/R injury [297]. Pancreatic islet‐derived miR‐136‐3p silences nardilysin convertase (NRDC) gene in skeletal muscle, promotes glycolytic metabolism, and supports myogenesis and glucose metabolism [298]. In the central nervous system, muscle‐derived miR‐17‐5p and miR‐20a‐5p can cross the BBB, activate hippocampal mTOR signaling, enhance synaptic plasticity, and alleviate cognitive impairment caused by chronic cerebral hypoperfusion [204]. Together, these findings show that miRNAs and exosomes provide targeted, tissue‐specific routes for exercise‐induced signaling.

#### 2.5.6 Other Exerkines

Apelin is a contraction‐induced myokine that contributes to bone–muscle crosstalk. It promotes osteoblast differentiation and mineralization and may cooperate with IGF‐1 and TGF‐β1 to support anabolic effects in bone and skeletal muscle, helping maintain bone remodeling balance [299]. Apelin also activates AMPK signaling to promote mitochondrial biogenesis and enhances glycolysis by increasing 6‐phosphofructo‐2‐kinase/fructose‐2,6‐bisphosphatase 3 (PFKFB3) and c‐MYC expression in endothelial cells, promoting angiogenesis [271, 300].

Follistatin is secreted by many tissues, including muscle, but is mainly produced in the liver [301, 302]. Studies have shown that loss of follistatin in muscle activates the MSTN/Activin A/Smads signaling pathway, which negatively affects muscle homeostasis and reduces exercise capacity [303]. Moreover, liver‐derived follistatin inhibits MSTN, activating mTORC1 signaling to increase energy expenditure in skeletal muscle, mediating metabolic crosstalk in the liver–muscle axis [304].

OCN is an osteoblast‐derived osteokine induced by exercise. UcOCN can cross the BBB, regulate neurotransmitter signaling, improve cognition, and reduce PD‐related neurotoxicity through AKT/GSK3β signaling [305]. Exercise‐induced ucOCN also stimulates adiponectin secretion, improving fat metabolism and systemic insulin sensitivity [306].

**FIGURE 5:**
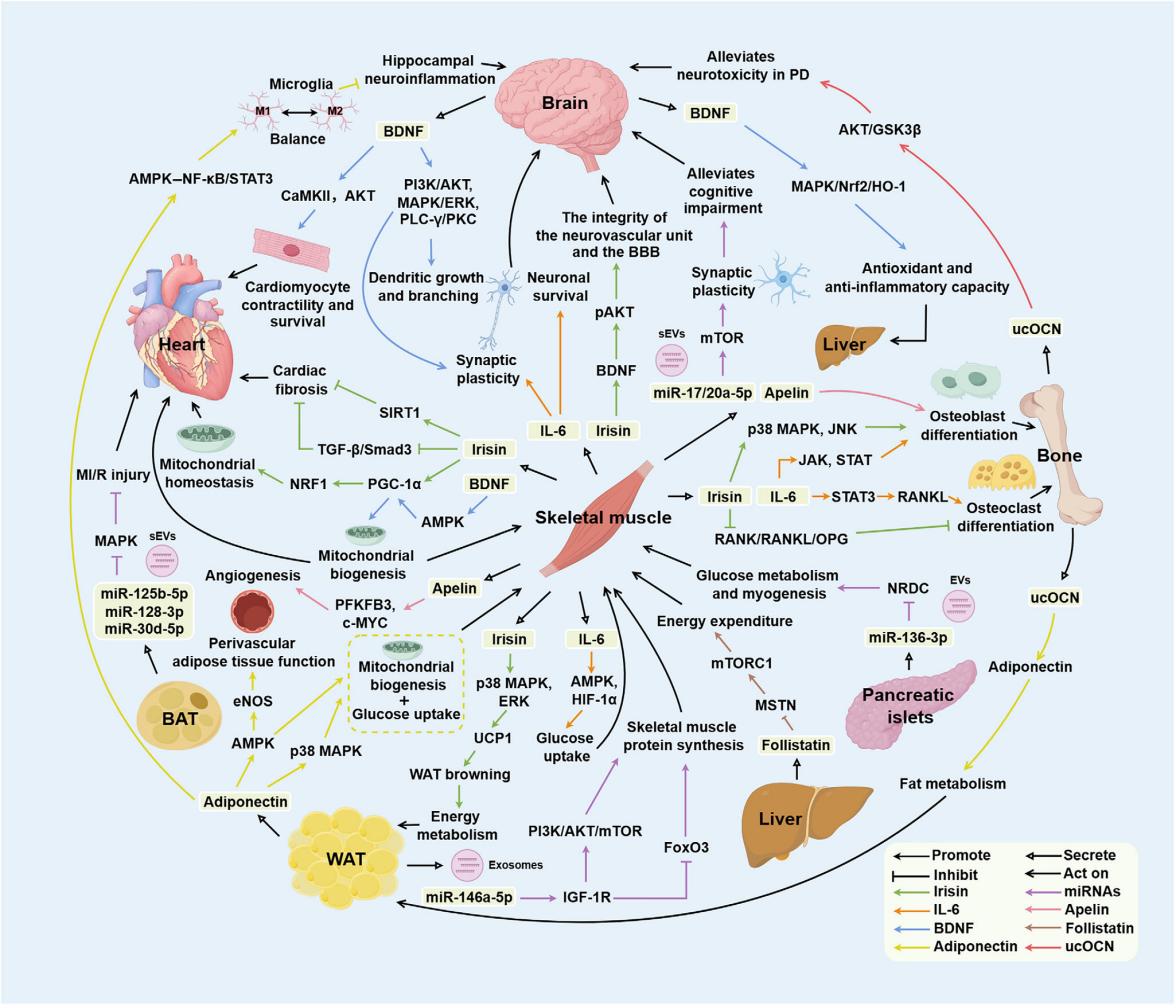
Exercise‐mediated cross‐system molecular network. This figure summarizes how key exercise‐induced factors coordinate multiorgan communication through interorgan signaling axes. In the skeletal muscle–bone axis, irisin promotes osteoblast differentiation by activating p38 mitogen‐activated protein kinase (p38 MAPK) and c‐Jun N‐terminal kinase (JNK) and suppresses receptor activator of nuclear factor‐kappa B (RANK)/RANKL/osteoprotegerin (OPG)‐mediated osteoclast differentiation. In addition, interleukin‐6 (IL‐6) regulates bone remodeling through Janus kinase (JAK) and signal transducer and activator of transcription (STAT) signaling, and also induces RANKL secretion via STAT3 signaling. In the skeletal muscle–heart axis, irisin reduces cardiac fibrosis by activating sirtuin 1 (SIRT1) and suppressing transforming growth factor beta (TGF‐β)/mothers against decapentaplegic homolog 3 (Smad3) signaling, and supports mitochondrial homeostasis by enhancing the transcriptional activity of peroxisome proliferator‐activated receptor gamma coactivator 1‐alpha (PGC‐1α) and nuclear respiratory factor 1 (NRF1). Brain‐derived neurotrophic factor (BDNF) activates AMP‐activated protein kinase (AMPK)/PGC‐1α signaling to support mitochondrial biogenesis, improving cardiac function. In the skeletal muscle–vascular axis, apelin promotes angiogenesis by increasing the levels of 6‐phosphofructo‐2‐kinase/fructose‐2,6‐bisphosphatase 3 (PFKFB3) and c‐MYC. In the skeletal muscle–brain axis, irisin upregulates cortical BDNF and phosphorylated protein kinase B (pAKT) expression, enhancing the integrity of the neurovascular unit and blood–brain barrier (BBB), while IL‐6 enhances neuronal survival and synaptic plasticity. Muscle‐derived microRNA‐17‐5p (miR‐17‐5p) and miR‐20a‐5p activate hippocampal mechanistic target of rapamycin kinase (mTOR) signaling to improve synaptic plasticity. In the skeletal muscle–adipose axis, irisin activates p38 MAPK and extracellular signal‐regulated kinase (ERK) to upregulate uncoupling protein 1 (UCP1) expression, inducing browning of white adipose tissue (WAT) and then enhancing energy metabolism. In the brain–heart axis, brain‐derived BDNF may enhance cardiomyocyte contractility and increase survival by activating calcium/calmodulin‐dependent protein kinase II (CaMKII) and protein kinase B (AKT). In the brain–liver axis, BDNF promotes MAPK/nuclear factor erythroid 2‐related factor 2 (Nrf2)/heme oxygenase‐1 (HO‐1) pathway, improving hepatic antioxidant and anti‐inflammatory capacity. In the adipose–skeletal muscle axis, adiponectin promotes skeletal muscle glucose uptake and mitochondrial biogenesis by activating AMPK and p38 MAPK. Adipose‐derived exosomal miR‐146a‐5p also targets insulin‐like growth factor 1 receptor (IGF‐1R) to activate phosphoinositide 3‐kinase (PI3K)/AKT/mTOR pathway or inhibit forkhead box O3 (FoxO3) signaling, promoting skeletal muscle protein synthesis. In the adipose–heart axis, brown adipose tissue (BAT)‐derived small extracellular vesicles (sEVs) deliver miR‐125b‐5p, miR‐128‐3p, and miR‐30d‐5p to the heart and reduce myocardial ischemia/reperfusion (MI/R) injury by inhibiting proapoptotic MAPK signaling. In the adipose–vascular axis, adiponectin improves vascular function by activating AMPK/endothelial nitric oxide synthase (eNOS) signaling to improve vascular health. In the adipose–brain axis, adiponectin activates the AMPK‐nuclear factor kappa‐light‐chain‐enhancer of activated B cells (NF‐κB)/STAT3 signaling to maintain the M1/M2 balance of microglia, suppressing hippocampal neuroinflammation. In the pancreatic islet–skeletal muscle axis, pancreatic islet‐derived miR‐136‐3p silences the nardilysin convertase (NRDC) gene to improve glucose metabolism and myogenesis in skeletal muscle. In the liver–skeletal muscle axis, liver‐derived follistatin inhibits myostatin (MSTN), activating mTORC1 signaling to increase energy expenditure in skeletal muscle. In the bone–brain axis, uncarboxylated osteocalcin (ucOCN) alleviates neurotoxicity in Parkinson's disease (PD) by activating the AKT/glycogen synthase kinase 3 beta (GSK3β) pathway. In the bone–fat axis, elevated ucOCN stimulates adiponectin secretion, improving fat metabolism. Local autocrine effects: BDNF activates pathways such as PI3K/AKT, MAPK/ERK, and phospholipase C gamma (PLCγ)/protein kinase C (PKC), promoting dendritic growth and branching of neurons and enhancing synaptic plasticity. IL‐6 promotes glucose uptake in muscle by activating AMPK‐ and hypoxia‐inducible factor 1‐alpha (HIF‐1α)‐dependent signaling. Together, these axes form an exerkine‐mediated molecular network through which exercise coordinates multiorgan adaptation.

As shown in Figure 5, the exerkines described above connect multiple systems through interorgan communication axes. Despite their different tissue origins, many converge on shared pathways such as AMPK and MAPK to support mitochondrial biogenesis, metabolic remodeling, and cell survival. They also suppress inflammation and oxidative stress, partly by inhibiting NF‐κB and activating Nrf2 signaling. These pleiotropic effects help explain how exercise coordinates multisystem remodeling through a limited set of signaling mediators.

### 2.6 Clinical Evidence of Exercise‐Mediated Multisystem Benefits

The preceding sections describe the molecular mechanisms by which exercise benefits multiple physiological systems and modulates related diseases. These effects are supported by a growing body of clinical evidence. This section summarizes representative studies from recent years covering multiple systems, including the musculoskeletal system (chronic low back pain [307], BMD [308], sarcopenia [309]), cardiovascular system (hypertension [310], heart failure with preserved ejection fraction [311]), nervous system (autism spectrum disorder [312, 313]), and metabolic system (T2DM [314], obesity [315]). These trials employed various exercise modalities (e.g., aerobic training, resistance training, and vibration training) and intervention durations (ranging from 8 to 52 weeks), systematically assessing improvements in physiological function, metabolic parameters, quality of life, and disease outcomes. Overall, clinical trials show that exercise‐induced adaptations improve not only target organ function but also systemic health, supporting exercise as a practical strategy for disease prevention and adjunctive treatment (Table 1).

**TABLE 1:** Clinical evidence of exercise‐mediated multisystem benefits.

Registration number and references	Target condition	Participants	Interventions	Outcomes
NCT05481996 [307]	Chronic LBP	47 participants 18–60 years old PSW with chronic LBP (numerical rating scale score ≥3/10)	Intervention group = an 8‐week mobile health‐based core training exercise and health education, *n* = 23 Control group = mobile health‐based health education, *n* = 24	Pain and disability ↓, work disability ↓ Quality of life ↑, self‐efficacy ↑ Anxiety symptom ↓ Depression, and stress symptoms − Sleep quality −
NCT02295072 [308]	Bone mineral density	77 participants Children aged 8–11 years with overweight or obesity 65% boys, 35% girls	The exercise intervention with a minimum of 3 sessions/week for 20 weeks (60 min aerobic + 30 min resistance), *n* = 38 The control group continued their usual routines, *n* = 39	Total body aBMD ↑ Legs aBMD ↑ Head, arms, lumbar spine, and pelvis aBMD −
ChiCTR2100051178 [309]	Sarcopenia in older adults	27 participants Older people with sarcopenia aged ≥65 years	The whole‐body vibration training group, *n* = 14 The resistance training group, *n* = 13 Both interventions consisted of 30‐min sessions, 3 times weekly over 12 weeks	KES ↑, HS ↑ Body composition (body weight, BMI, PBF)↓ ASMI ↑ Physical performance (GS ↑, 5CST ↓, and SPPB ↑) Blood biomarkers (IGF‐1 ↑, growth hormone ↑, FST ↓ and CK ↑) Quality of life (SF‐36) ↑
NCT04275037 [310]	High normal blood pressure or Stage I hypertension	76 participants Aged 65 years or older with high normal BP or Stage I hypertension	An 8‐week home‐based isometric handgrip training, *n* = 27 An 8‐week home‐based aerobic exercise training, *n* = 26 Usual medical care plus lifestyle advice, *n* = 23	Office blood pressure ↓ Ambulatory blood pressure −
ISRCTN86879094 [311]	HFpEF	322 participants HFpEF patients with an average age of 70 years 192 females, 130 males 86% NYHA class II 99% Caucasian	A 12‐month combined endurance and resistance training, *n* = 161 Usual care, *n* = 161	V̇O_2peak_ ↑ NYHA class ↓ Other secondary endpoints (diastolic function, GSA, time to cardiovascular hospitalization or all‐cause mortality) of modified Packer score −
ChiCTR2400087262 [312]	ASD	50 participants Chinese Han children aged 3–10 years diagnosed with moderate to severe ASD	The exercise intervention = a 12‐week ball combination training program with five 45‐min sessions each week The control group	The motor ability including manual dexterity, aiming and catching, and balance (MABC‐2) ↑
ChiCTR2400087989 [313]	Moderate autism	25 participants First‐grade children with moderate autism	The fundamental movement skills intervention for 18 weeks, with sessions held four times per week, each lasting 45 min at a moderate intensity, *n* = 13 The control group = regular daily routine, *n* = 12	Locomotor skills (TGMD‐3 scores) ↑ Executive function (BRIEF scores) ↑ Social interaction ability (SRS‐2 scores) ↑
ChiCTR2500104389 [314]	T2DM	209 participants Adults aged 18–70 years with T2DM lasting at least 1 year, and meeting the obesity criterion defined by Chinese standards (BMI ≥ 28 kg/m^2^)	The digital outdoor exercise group received a 12‐week smartphone‐facilitated intervention: 150 min/week of moderate‐intensity outdoor aerobic exercise plus two weekly resistance sessions. The clinic‐based exercise program group = three 60‐min sessions per week of moderate‐intensity aerobic and resistance exercise	Glycemic control ↑ (HbA1c ↓) Anthropometric measures (BMI, waist circumference) ↓ Cardiovascular measures (resting systolic and diastolic blood pressure, resting heart rate) ↓ Glycemic and metabolic measures (fasting plasma glucose, fasting insulin, HOMA‐IR, triglycerides) ↓ Physical function measures (6‐min walk test distance, 30‐s chair‐stand test) ↑ Patient‐reported outcomes (SF‐36 PCS, SF‐36 MCS) ↑
NCT04122716 [315]	Obesity	193 participants Adults with obesity (age 18–65 years, body mass index 32–43 kg/m^2^) without diabetes mellitus	The placebo + exercise group followed a 52‐week program including a 6‐week ramp‐up, and then required at least 150 min/week of moderate intensity, 75 min/week of vigorous intensity, or an equivalent combination. The liraglutide + usual physical activity group = 0.6 mg once daily with weekly increments of 0.6 mg until 3.0 mg was reached. The liraglutide + exercise group The control group = placebo + usual physical activity	Physical functional performance test: time to complete the stair climb test (seconds) ↓, self‐reported physical functioning − Cardiorespiratory fitness test: V̇O_2peak_ relative to FFM (mL/min/kg FFM), V̇O_2peak_ in absolute values, V̇O_2peak_ normalized to body weight, peak power output during the cycle test (exercise tolerance) ↑ Muscle strength test: knee extensor peak torque (Nm) −, knee extensor peak torque normalized to body weight ↑

*Abbreviations*: ↑, increase; ↓, decrease; −, no significant change; LBP, low back pain. PSW, public safety workers; aBMD, areal bone mineral density; KES, knee extension strength; HS, handgrip strength; BMI, body mass index; PBF, percentage of body fat; ASMI, appendicular skeletal muscle mass index; GS, gait speed; 5CST, 5‐time chair stand test; SPPB, short physical performance battery; IGF‐1, insulin‐like growth factor 1; FST, follistatin; CK, creatine kinase; SF‐36, Short Form‐36 Health Survey; HFpEF, heart failure with preserved ejection fraction; NYHA, New York Heart Association; V̇O_2peak_, peak oxygen consumption; GSA, global self‐assessment; ASD, autism spectrum disorder; MABC‐2, Movement Assessment Battery for Children‐Second Edition; TGMD‐3, Test of Gross Motor Development‐Third Edition; BRIEF, Behavior Rating Inventory of Executive Function; SRS‐2, Social Responsive Scale‐Second Edition; T2DM, Type 2 diabetes mellitus; HbA1c, glycated hemoglobin; HOMA‐IR, Homeostasis Model Assessment of Insulin Resistance; PCS, physical component score; MCS, mental component summary; FFM, fat‐free mass.

## 3 Exercise‐Mediated Molecular Networks In Aging

Aging is a progressive decline in function across multiple organs and systems [316]. Exercise has received sustained attention in antiaging research due to its capacity to simultaneously target multiple aging‐related hallmarks [317].

### 3.1 Molecular Mechanisms of Exercise in Aging Regulation

#### 3.1.1 Regulation of Telomere Maintenance and Stability

Exercise may contribute to telomere maintenance during aging. It does not directly lengthen telomeres, but may slow telomere erosion by improving the internal environment, regulating enzymatic activity, and reducing psychological stress [318]. Chronic inflammation and oxidative stress accelerate telomere attrition [319]. Regular exercise can reduce low‐grade inflammation, modulate cytokines such as tumor necrosis factor‐alpha and IL‐6 [320], and activate the Nrf2‐mediated antioxidant defense system to create a redox‐balanced environment to mitigate telomere erosion [321].

Telomerase helps limit telomere attrition. Exercise activates the AMPK/PGC‐1α/NRF1 axis, supporting mitochondrial and metabolic adaptation, and may also regulate telomere‐associated RNAs such as telomeric repeat‐containing RNA (TERRA) [322]. Psychological status also modulates telomere length. Exercise may mitigate stress‐associated neuroendocrine activation, partly through modulation of cortisol dynamics and psychological well‐being, thereby indirectly protecting telomere stability [323, 324].

As a central hallmark of cellular senescence, telomere length governs cellular function and proliferative potential [325, 326]. Therefore, telomere maintenance may have broad systemic effects. In the immune system, preserved telomere length may delay T‐cell and macrophage senescence, sustaining immune diversity [327, 328]. In metabolism, telomere integrity supports pancreatic β‐cell insulin secretion and hepatocyte regeneration [329, 330]. In skeletal muscle, telomere maintenance in satellite cells is associated with preserved regenerative potential and slower sarcopenia progression [331].

**FIGURE 6:**
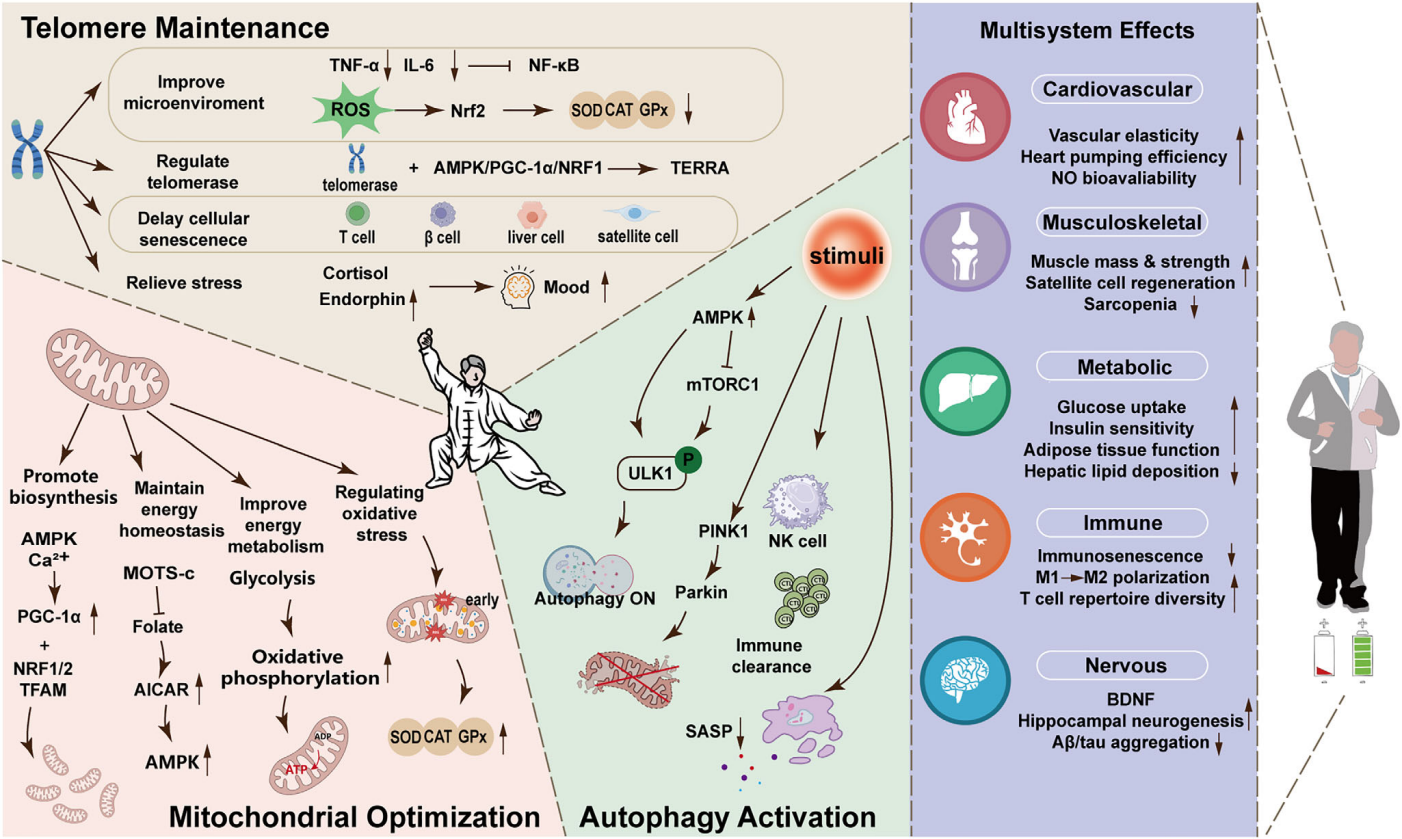
Exercise counteracts aging through telomere maintenance, mitochondrial optimization, and autophagy activation. This figure summarizes three major antiaging mechanisms induced by exercise: telomere maintenance, mitochondrial optimization, and autophagy activation. Exercise supports telomere stability by improving the cellular microenvironment, regulating telomerase activity, reducing stress‐related damage, and delaying cellular senescence. It also activates AMP‐activated protein kinase (AMPK), which promotes mitochondrial biogenesis through the peroxisome proliferator‐activated receptor gamma coactivator 1‐alpha (PGC‐1α)/nuclear respiratory factor 1/2 (NRF1/2)/mitochondrial transcription factor A (TFAM) axis. The mitochondria‐derived peptide mitochondrial open reading frame of the 12S rRNA‐c (MOTS‐c) may further support energy homeostasis through the folate‐5‐aminoimidazole‐4‐carboxamide ribonucleotide (AICAR)–AMPK pathway. Exercise improves mitochondrial quality by enhancing oxidative phosphorylation and increasing antioxidant enzymes, including superoxide dismutase (SOD), catalase (CAT), and glutathione peroxidase (GPx). It also activates autophagy through AMPK‐dependent phosphorylation of unc‐51‐like kinase 1 (ULK1) and relief of mechanistic target of rapamycin complex 1 (mTORC1)‐mediated inhibition. Exercise‐induced mitophagy, involving the PTEN‐induced kinase 1 (PINK1)–Parkin pathway, promotes clearance of dysfunctional mitochondria. Autophagy also reduces the senescence‐associated secretory phenotype (SASP) and facilitates removal of damaged cellular components. These coordinated effects support healthier aging across cardiovascular, musculoskeletal, metabolic, immune, and nervous systems.

Overall, exercise may support telomere stability through anti‐inflammatory and antioxidant effects, telomerase regulation, and stress reduction. These adaptations may help interrupt inflammation‐driven senescence and establish a beneficial cascade that preserves muscle mass and strength, improves cardiovascular function, and enhances systemic oxygen utilization [332, 333]. Thus, exercise‐mediated telomere protection may contribute to coordinated antiaging adaptations across multiple organ systems (Figure 6).

#### 3.1.2 Mitochondrial Biogenesis and Quality Control

Mitochondria are central to cellular energy metabolism and also regulate apoptosis, oxidative stress, and calcium homeostasis [334]. During aging, mitochondrial biogenesis declines, largely because of reduced PGC‐1α signaling, leading to fewer functional mitochondria and impaired energy metabolism.

Exercise activates AMPK and Ca^2+^‐dependent signaling to upregulate PGC‐1α, initiating mitochondrial DNA replication and respiratory chain complex assembly, thereby enhancing mitochondrial network density [335]. Activated PGC‐1α coordinates with NRF1/2 and mitochondrial transcription factor A (TFAM) to drive mitochondrial biogenesis [336]. Exercise may also increase circulating the mitochondria‐derived peptide mitochondrial open reading frame of the 12S rRNA type C (MOTS‐c), a mitochondria‐derived peptide involved in energy homeostasis through the folate/5‐aminoimidazole‐4‐carboxamide ribonucleotide (AICAR)/AMPK pathway [231]. Beyond mitochondrial biogenesis, exercise also improves substrate utilization and redox balance. It enhances metabolic flexibility and oxidative phosphorylation, increasing ATP production efficiency [337], and upregulates transporters for glucose, lactate, and fatty acids to support more efficient substrate use [231]. Moderate‐intensity continuous training increases mitochondrial content, Complex I activity, and proteins involved in the tricarboxylic acid cycle and oxidative phosphorylation [337]. These metabolic changes are accompanied by adaptive ROS signaling, with early‐phase ROS triggering adaptive responses and long‐term training upregulating antioxidant enzymes such as SOD, CAT, and GPx [338, 339]. Regular exercise also activates Nrf2‐mediated antioxidant defense and helps limit oxidative damage to mitochondrial DNA [340].

These adaptations improve function across multiple systems. In skeletal muscle, exercise increases mitochondrial number and function, improving muscle strength and endurance [341, 342]. In the brain, exercise‐induced BDNF and improved systemic metabolism support mitochondrial health and synaptic plasticity [58]. In the immune system, optimized mitochondrial function drives macrophage polarization toward an anti‐inflammatory M2 phenotype, reducing systemic inflammation [343, 344]. In the cardiovascular system, exercise improves mitochondrial function in endothelial cells and cardiomyocytes, increases NO bioavailability, and enhances cardiac pumping efficiency [345, 346].

In summary, exercise promotes mitochondrial quality control and metabolic resilience through coordinated regulation of biogenesis, substrate utilization, and redox homeostasis, contributing to systemic antiaging adaptations (Figure 6).

#### 3.1.3 Induction and Regulation of Autophagy

Autophagy maintains protein homeostasis by clearing damaged proteins and dysfunctional organelles. Regular exercise activates autophagic pathways, modulates senescence‐associated phenotypes, and may enhance immune‐mediated clearance of senescent cells, thereby supporting tissue homeostasis [347, 348].

Exercise activates autophagy via energetic and mechanical stress. ATP depletion activates AMPK, which phosphorylates ULK1 to trigger autophagy [349, 350]. Concurrently, exercise inhibits mTORC1, relieving its suppression of autophagy [351]. Aerobic exercise may maintain mitochondrial homeostasis through AMPK activation, mTORC1 inhibition, and possible FOXO3a involvement [352].

Mitophagy removes dysfunctional mitochondria and is essential for mitochondrial quality control, with the PINK1/Parkin pathway as a key regulator [353]. Resistance training has been associated with activation of this pathway and improved mitochondrial function in animal models [354]. Exercise may also strengthen immune surveillance by promoting natural killer (NK) cell‐ and cytotoxic T cell‐mediated clearance of senescent cells, preserving T cell diversity, and reducing the senescence‐associated secretory phenotype (SASP) [344, 355, 356].

By regulating autophagy and senescent cell clearance, exercise may protect multiple organ systems. In skeletal muscle, autophagic removal of damaged mitochondria helps restore stem cell regenerative potential and improve muscle quality and metabolism [357]. In the brain, autophagy may clear misfolded proteins such as Aβ and tau, while supporting hippocampal neurogenesis and cognitive resilience [58]. In the cardiovascular system, mitophagy helps remove damaged mitochondria from ischemic cells and may reduce apoptosis, limiting heart failure progression and atherosclerotic remodeling [347, 358]. In the vasculature and liver, autophagy promotes lipid degradation and supports vascular elasticity and metabolic function [359, 360]. Together, these effects suggest that exercise helps preserve tissue homeostasis and provides broad antiaging protection (Figure 6).

### 3.2 Mechanistic Gaps and Translational Hurdles in Exercise‐Mediated Aging Regulation

Although exercise supports healthy aging through telomere protection, mitochondrial optimization, and autophagy activation, important gaps remain. The dose–response relationships between exercise type, intensity, frequency, and molecular adaptation are still unclear. Notably, exercise‐driven telomerase activation, ROS signaling regulation, and mitophagy induction follow inverted U‐shaped or threshold patterns [318, 361, 362]. Several mechanistic issues also require clarification, including differences in telomere protection between stem and somatic cells [363], cell‐type‐specific autophagy responses to exercise, the long‐term effects of senescent cell clearance, and heterogeneous responses among cellular subtypes [364]. Moreover, many mechanistic insights are derived predominantly from animal or short‐term intervention studies, whereas their long‐term translatability to human aging remains incompletely established. At the individual level, genetic background (e.g., PGC‐1α polymorphism), sex, and age drive marked variability in exercise responsiveness [365, 366]. Given that exercise–responsive pathways are highly interconnected, future studies should use systems biology to map aging‐related networks, define adaptive thresholds, and evaluate precision exercise prescriptions in diverse populations, especially older adults.

## 4 Molecular Adaptations To Exercise In Extreme Environments

Most known benefits of exercise are based on normoxic, normobaric, and normal‐gravity conditions. Under extreme environments, such as microgravity, hyperbaric exposure, or hypoxia, physiological stress thresholds shift and exercise adaptations may change. This section discusses how these environments affect the cardiovascular, musculoskeletal, metabolic, and immune systems, and how targeted exercise training may help maintain multisystem homeostasis.

### 4.1 Microgravity: Cellular Sensing and Atrophy Signaling

Prolonged spaceflight induces cardiovascular deconditioning [367], bone loss, muscle atrophy, and immune suppression [368, 369, 370]. Exercise is a key countermeasure against these effects. International space station (ISS)‐based nonrandomized studies suggest that combined aerobic and resistance training helps preserve vascular tone and baroreflex function [371], whereas high‑intensity interval training increases cardiac output and alleviates cardiac atrophy [372, 373]. Resistance training with the advanced resistive exercise device (ARED) helps limit microgravity‐induced muscle and bone loss, and alendronate combined with ARED‐based exercise may further slow the decline in areal BMD after 6‐month ISS missions [374]. Spaceflight also impairs T‑cell and NK cell function, elevates salivary stress hormones, and reactivates latent herpesviruses [375]. Ground‐based head‐down bed rest studies suggest that moderate aerobic exercise can improve NK cell activity, T‐cell ratios, and simulated microgravity‐induced immune dysfunction [376]. However, current evidence remains limited by small‐sample human studies, baseline interindividual variability, few randomized controlled trials, and reliance on ground‐based simulations, which limits generalizability.

### 4.2 High‐Pressure: Cellular Stress Responses

Deep‐sea hyperbaric environments affect the body through high pressure, low temperature, and breathing restriction, disturbing hematopoietic, vascular endothelial, and neuroendocrine function [377]. Exercise preconditioning may help reduce these effects. Hyperbaric exposure can induce erythrocytopenia, endothelial dysfunction, and oxidative stress. In 21 male divers exposed once to 18–20 m depth, platelet counts decreased and serum VCAM‐1 increased, indicating endothelial injury [378, 379, 380]. Exercise before diving can reduce venous bubble formation after decompression. A randomized crossover trial in 12 divers showed that one session of high‐intensity interval exercise 24 h before diving reduced bubble formation [381]. In military divers, 45 min of running at 60–80% maximal heart rate 2 h before diving lowered the bubble grade from 1.25 to 0.44, with no participant showing an increase compared with the nonexercise condition [382]. Moreover, hyperbaric stress may also disrupt the hypothalamic–pituitary–gonadal axis and cortisol–testosterone balance, contributing to muscle atrophy and strength loss [383], whereas periodic resistance training may help preserve this balance and reduce muscle breakdown [384, 385]. Current evidence is still limited by the lack of randomized controlled trials in hyperbaric settings and by differences between animal protocols and real‐world diving conditions.

### 4.3 Hypoxia: Oxygen‐Sensing and Metabolic Adaptation

High‐altitude hypoxia impairs physiological function by reducing oxygen delivery and damaging mitochondrial function [386, 387, 388, 389, 390, 391]. Animal studies suggest that high‑intensity interval training upregulates PGC‑1α to promote mitochondrial biogenesis [392, 393]. At an altitude of 5000 m, maximal oxygen consumption declines and myofiber types shift, leading to muscle fatigue, atrophy, and dysfunction [394]. Long‑term high‑altitude exposure can cause chronic mountain sickness (CMS), often accompanied by pulmonary hypertension and heart failure [389]. Moderate‐intensity aerobic training has been reported to reduce hematocrit and relieve CMS symptoms in native Andean highlanders [395]. But the study included only young men. Further work is needed in women, older adults, and other CMS populations. Current evidence is limited by differences between hypoxic chamber models and real high‐altitude environments, limited data from long‐term high‐altitude residents, and insufficient subgroup analyses by sex, age, residence duration, and genetic background.

### 4.4 Clinical and Occupational Implications

Exercise may help protect cardiovascular and musculoskeletal function in extreme environments, such as microgravity, hyperbaria, and hypoxia, by regulating cellular stress responses, metabolic adaptation, and immune homeostasis. These findings are important for protecting astronauts, divers, and high‐altitude workers and may also reveal basic mechanisms of biological adaptation and decompensation. However, translation to real‐world settings requires caution. Controlled experimental conditions may not fully reflect occupational environments, intervention safety must be tested in specific populations, and individual responses can vary substantially. Personalized exercise strategies may therefore be needed. Overall, extreme environments offer a valuable model for studying the limits of exercise‐induced molecular adaptation, while underscoring the need for further integrative research.

## 5 Precision Exercise And Molecular Signatures

Marked variability in exercise response has promoted the development of precision exercise medicine. Precision health aims to optimize interventions by integrating genetic, physiological, behavioral, and environmental factors. This chapter discusses the basis of exercise–response variability, the role of multiomics and AI in individualized exercise prescription, and the progress and challenges of exercise mimetics.

### 5.1 Determinants of Exercise Responsiveness

Exercise prescription is shifting away from a “one‐size‐fits‐all” approach. Individuals vary widely in their responses to the same exercise program, from “high responders” with clear benefits to “low responders” with limited improvement [396]. True “nonresponders” remain debated, as apparent nonresponsiveness may reflect insufficient dose, poor adherence, or insensitive outcome measures. This variability is influenced by genetics and epigenetics [199], as well as lifestyle factors, aging, medication use, and the gut microbiome. Defining these determinants is central to personalized exercise prescription [397].

#### 5.1.1 Genetic Polymorphisms

Genetic polymorphisms, especially single nucleotide polymorphisms (SNPs), contribute to differences in exercise response. The angiotensin‐converting enzyme insertion/deletion (ACE I/D) polymorphism has been associated with endurance‐ and power‐related traits [398], while the alpha‐actinin‐3 (ACTN3) R577X (XX) genotype, marked by α‐actinin‐3 deficiency and more oxidative muscle metabolism, may favor endurance phenotypes but is less advantageous for elite power performance [399]. Variants in PPARα and PPARGC1A may also affect aerobic trainability [235, 400]. For health outcomes, carriers of the fat mass and obesity‐associated protein obesity‐risk allele often show greater weight loss and metabolic improvement after exercise, suggesting that exercise may partly offset genetic obesity risk [401, 402]. However, SNPs are static and explain only part of response variability, making epigenetic mechanisms important for understanding dynamic exercise adaptation [403, 404].

#### 5.1.2 Epigenetic Modifications

Epigenetic mechanisms shape exercise‐induced gene expression and may serve as biomarkers of training adaptation [403]. Exercise has been associated with ten‐eleven translocation‐mediated DNA demethylation, including promoter demethylation of metabolic and inflammatory genes such as PPARGC1A [405, 406]. Baseline methylation patterns may help predict exercise responsiveness, while training‐induced methylation changes reflect adaptive remodeling [407, 408]. Histone modifications regulated by histone acetyltransferases and HDACs alter chromatin accessibility and support exercise‐induced cellular adaptation [409, 410]. Circulating miRNAs are being explored as minimally invasive biomarkers of exercise adaptation [298, 411], and baseline miRNA profiles have been associated with predisposition to endurance versus strength performance [412].

### 5.2 Molecular‐Guided Exercise Prescription

Exercise responsiveness is complex and cannot be fully captured by conventional phenotyping alone. Integrating multiomics with AI offers a framework for linking molecular profiles to individualized exercise prescriptions. Multiomics can define multidimensional response patterns [10]; machine learning (ML) can identify response subtypes and predict intervention efficacy [413]; and digital twins combined with longitudinal biomarkers may support adaptive prescription refinement and monitoring [414]. Despite challenges in data standardization and clinical validation, this framework may help move exercise medicine from experience‐based recommendations toward data‐driven precision practice.

#### 5.2.1 Omics and AI in Exercise

Exercise science is increasingly converging with data science to understand the systemic and nonlinear nature of exercise responses [415]. Multiomics enables unbiased profiling across biological layers, while AI and ML extract patterns from large datasets and build predictive models. Together, these tools support more individualized and evidence‐based exercise interventions [416, 417, 418].

Multiomics captures exercise responsiveness from genetic variation and epigenetic regulation to gene expression, proteins, and metabolites [419]. Genomics and epigenomics help explain interindividual variability [404, 420]. Transcriptomics identifies exercise‐sensitive mRNAs and noncoding RNAs [421, 422], and proteomics and metabolomics reveal functional effectors, novel exerkines such as Lac–Phe, and dynamic metabolic remodeling [420, 423, 424]. Bioinformatic integration then links these molecular layers into networks that connect genotype with exercise–responsive phenotypes [10, 425].

AI and ML are well suited to analyzing high‐dimensional multiomics data [426, 427]. Unsupervised methods, such as clustering, can stratify participants by molecular profiles and identify exercise–response subtypes [428]. Supervised models use baseline multiomics, clinical, and genetic data to predict individual outcomes, such as changes in VO_2_max, and to classify high and low responders [429, 430]. Algorithms such as least absolute shrinkage and selection operator can identify compact biomarker panels with better predictive value and lower cost [431], while deep learning can integrate medical images, wearable time‐series data, and multiomics profiles to build dynamic personalized health models [432, 433].

Combining multiomics with AI may enable in silico trials, adaptive exercise prescriptions, and digital twin models that simulate individual responses [414, 434, 435]. These tools could advance predictive, preventive, personalized, and participatory (4P) medicine [436]. AI‐driven drug discovery may also help design exercise mimetics, such as irisin‐like molecules, or identify targets that shape exercise responsiveness [10, 437].

#### 5.2.2 Biomarker‐Based Monitoring and Clinical Translation

A major challenge in precision health is identifying biomarkers that dynamically reflect physiological status and exercise adaptation. Traditional measures such as body mass index and blood pressure often change slowly and may miss early subclinical responses [438]. Circulating miRNA‐based “health scores” may provide broader molecular readouts of adaptation [439, 440], while EVs, metabolites such as branched‐chain amino acids, and inflammatory markers such as high‐sensitivity C‐reactive protein (hs‐CRP) are also being explored for monitoring exercise responses [441, 442, 443]. Integrating these markers into multiparametric models may improve physiological assessment and support adaptive exercise prescription.

Multiomics and AI are moving exercise prescription toward precision strategies, but several barriers remain. Standardized exercise phenotyping, harmonized molecular panels, and cross‐platform data integration are needed to improve reproducibility [444]. AI models also require stronger generalizability and clinical validation, and causal mediators must be distinguished from correlated molecular signatures. Approaches such as nested cross‐validation, transfer learning, federated learning, and explainable AI may improve robustness and interpretability, but prospective biomarker‐stratified trials are needed to test whether AI‐assisted prescriptions outperform conventional strategies [445]. Early studies suggest that integrating molecular signatures, functional phenotyping, and digital monitoring may help stratify training responsiveness in older adults with sarcopenia [446, 447], and similar precision frameworks using continuous glucose monitoring, multiomics, inflammatory markers, and wearable data are being explored in T2DM [448].

In the future, closed‐loop systems combining baseline multiomics, AI‐guided prescriptions, and real‐time feedback may enable adaptive exercise optimization, while digital twin models may support virtual prediction of intervention responses. Despite challenges such as algorithmic bias, regulatory approval, feasibility, and cost‐effectiveness, continued advances are making precision exercise medicine increasingly translatable.

### 5.3 Exercise Mimetics: Targeting Molecular Pathways

Insights from precision exercise biology and molecular profiling have accelerated the identification of candidate exercise‐mimetic targets. Although exercise is essential for preventing and managing chronic diseases, people who may benefit most, such as those with advanced heart failure, severe arthritis, or frailty, often cannot exercise adequately [449, 450, 451]. Exercise mimetics, often described as “exercise pill”‐like strategies, aim to reproduce selected molecular effects of exercise and may offer a translational option for people who cannot engage in sufficient physical activity [452, 453].

#### 5.3.1 Design and Strategies of Exercise Mimetics

Exercise mimetics aim to reproduce selected exercise‐induced pathways and benefits, mainly through pathway agonists or exercise‐derived factors and their analogs [454]. Small‐molecule pathway agonists activate exercise–responsive signaling. AMPK agonists such as AICAR are converted to the AMP mimetic ZMP and activate AMPK, increasing mitochondrial content, fatty acid oxidation, and endurance in preclinical studies [455, 456]. PPARδ/β agonists such as GW501516 mimic exercise‐induced transcriptional programs and promote oxidative fiber remodeling, but their development was largely halted after carcinogenicity was reported in rodents [457, 458]. Sirtuin activators such as resveratrol may modulate SIRT1‐related metabolic pathways, although their specificity and efficacy remain controversial [459]. The strategy centered on exercise‐derived factors and their analogs has attracted considerable interest, focusing on directly using or engineering long‐acting analogs to replicate key exercise‐induced signaling molecules.

#### 5.3.2 Challenges and Future Directions

Exercise mimetics have therapeutic potential but face major safety and delivery challenges. Beyond the carcinogenic concerns reported for GW501516 [458], sustained pharmacological activation of pathways such as AMPK may cause cardiac or metabolic adverse effects in some preclinical models, while resveratrol shows limited specificity and broad off‐target activity [460, 461, 462]. Because targets such as AMPK and PPARδ regulate fundamental physiological processes, these agents may have narrow therapeutic windows [463, 464]. Protein‐ and peptide‐based mimetics face additional delivery barriers; for example, BDNF has poor bioavailability and limited BBB penetration, whereas metabolites such as Lac–Phe may have stability and pharmacokinetic limitations [222, 465]. Strategies such as PEGylation, nanoparticle encapsulation, viral delivery, and prodrug design are being explored to improve delivery [465]. Structurally optimized AMPK agonists, oral irisin mimetics, and selective HDAC3 inhibitors have shown promising preclinical potential, supporting further development of next‐generation exercise mimetics [460, 466].

In summary, exercise mimetics remain in an early stage, and whether they can fully reproduce the systemic, adaptive, and context‐dependent benefits of exercise is unclear. Still, as exercise‐related molecular networks and delivery technologies advance, safe and effective mimetics for people unable to exercise remain an important translational goal.

## 6 Conclusions And Perspectives

Exercise represents not merely energy expenditure, but a systemic “signal intervention.” By activating evolutionarily conserved biological programs, exercise reprograms organismal function at the molecular level, conferring enhanced resilience against disease and aging, thus representing an indispensable component of preventive medicine and healthy aging strategies. Herein, we establish an integrative framework for the systemic regulation induced by exercise across multiple dimensions, including multisystem signaling pathways, cross‐system coordinated regulation, adaptation to extreme environments, and precision exercise medicine. We summarize the molecular mechanisms of exercise‐induced health benefits and disease modulation across the musculoskeletal, cardiovascular, nervous, and metabolic–immune systems. Cross‐system actions of key exerkines reveal that the benefits of exercise arise from complex interaction networks involving multiple factors and multiple systems. Additionally, the regulatory roles of exercise in telomere homeostasis, mitochondrial function, and autophagy collectively form the biological basis against age‐related decline. Furthermore, the exercise‐induced adaptive regulation in extreme environments provides a unique perspective on the dynamic interplay between exercise and the environment.

Future studies should move beyond descriptive observations toward mechanistic understanding and personalized application. Integrating multiomics technologies with AI will help reveal dynamic molecular networks and further clarify the molecular basis of exercise‐induced adaptations. Multitissue integration of time‐series transcriptomics, proteomics, metabolomics, lipidomics, epigenomics, and immunomics may provide comprehensive insights into how exercise regulates immune, metabolic, and mitochondrial pathways [467]. At the same time, advances in AI offer new opportunities for integrated analysis of large‐scale multiomics datasets [468, 469, 470]. For example, the Multidimensional Modeling to Maximize Adaptations to exercise (M^3^AX) trial [471] combined multiomics profiling with deep learning to predict individual variability in exercise responses, providing an important basis for the development of precision exercise medicine.

Application of multiomics technologies can identify key signaling molecules and regulatory hubs in response to exercise, thereby providing potential targets for the development of exercise mimetics. As an alternative strategy for individuals unable to exercise, exercise mimetics may have important clinical applications. Recent progress has been made in the development of AMPK [456], PPARδ [457], SIRT1 [472], and other agonists, although major challenges remain, including recapitulating the complexity of exercise networks [473], safety risks [474], and improving drug delivery systems [475]. Future research should integrate multiomics approaches with AI‐based analyses to reveal the common biological basis underlying the systemic benefits of exercise and to explore combination therapies or multitarget mimetics. Additionally, establishing rigorous preclinical safety evaluation systems and developing novel drug delivery systems [475] are urgent issues that need to be addressed.

For both exercise and pharmacological interventions, accurate assessment and optimization require objective physiological and behavioral data. The integration of wearable devices and smart sensors with AI enables real‐time monitoring and dynamic feedback on physiological status, movement posture, and rehabilitation progress, thereby enhancing the precision and effectiveness of exercise interventions [476, 477, 478]. In musculoskeletal disorders, ML models can extract movement features and spinal profiles from motion videos of patients with nonspecific low back pain to accurately identify motor control impairment phenotypes, demonstrating the potential of AI to support personalized exercise rehabilitation [479]. However, the application of AI in exercise medicine still faces multiple challenges, including data heterogeneity [480], insufficient model generalizability [481], lack of interpretability [482], and ethical concerns [483]. Future efforts should focus on establishing standardized shared databases [484, 485], developing AI interpretability frameworks [486], and strictly validating model efficacy through multicenter clinical trials [487]. Further, AI should be combined with dynamic sensors to construct an individual‐level digital twin model, achieving truly precise exercise prescriptions.

Taken together, our review expands the understanding of the molecular mechanisms by which exercise confers systemic health benefits and outlines future directions for multiomics and AI‐driven precision exercise medicine as well as exercise mimetics. In the future, the deep integration of multiomics, AI, and individual data will promote the construction of a digital health ecosystem, transforming exercise into an accessible and efficient precision health tool capable of advancing global health and promoting healthy aging.

## Author Contributions

Shukuan Ling, Jianmin Wu, and Yi Wang conceived the topic and designed the framework of this review. Peifeng Ying, Huilin Wang, and Xiu Lu performed the literature search, wrote the manuscript, and prepared the figures and tables. Wenjuan Zhang, Zhehao Lin, Kai Wang, Shangxuan Li, Mingchao Jiang, Lu Chen, and Sarkawt Hamad organized the references and proofread the manuscript. Shukuan Ling, Jianmin Wu, and Yi Wang revised the manuscript and gave final approval of the submitted manuscript. All authors have read and approved the final manuscript.

## Funding

This research was supported by the National Natural Science Foundation of China (grant 82471913 to Shukuan Ling, 82572147 to Jianmin Wu), Zhejiang Provincial Natural Science Foundation of China (grant LZ23H210001 to Shukuan Ling), and Oujiang Laboratory (grant OJQD2022012 to Shukuan Ling).

## Conflicts of Interest

The authors declare no conflicts of interest.

## Ethics Statement

The authors have nothing to report.

## Data Availability

The authors have nothing to report.
